# Life lost due to the COVID-19 pandemic: A model-based cohort analysis of mortality displacement in the registered population of England

**DOI:** 10.1371/journal.pone.0348575

**Published:** 2026-05-08

**Authors:** Andrew Hughes, Sharmani Barnard, Clarissa Bauer-Staeb, Richard Holleyman, Samantha Dunn, Paul Fryers, John N. Newton, Justine Fitzpatrick, Paul Burton, Peter Goldblatt

**Affiliations:** 1 Office for Health Improvement and Disparities, London, United Kingdom; 2 School of Population Health, Curtin University, Bentley, Western Australia, Australia; 3 UK Health Security Agency, UK, Wellington House, London, United Kingdom; 4 Population Health Sciences Institute, Newcastle University, Newcastle upon Tyne, United Kingdom; 5 European Centre for Environment and Human Health, University of Exeter Medical school, Penryn, Cornwall, United Kingdom; 6 UCL Institute of Health Equity, Department of Epidemiology & Public Health, University College London, London, United Kingdom; Centers for Disease Control and Prevention, UNITED STATES OF AMERICA

## Abstract

**Background:**

Uncertainty about the prior health status of those dying during the pandemic has fuelled debate about its impact. To date, attempts to quantify life years lost during the pandemic have relied on using life tables without taking into account varying levels of vulnerability among those that died.

**Methods:**

Using retrospective, linked data from March 2020 to September 2022 for the cohort of all individuals in England alive at outset, we quantified the risk of death, associated with a wide variety of comorbidities, using primary care and hospital data, as well as evidence of vaccination and COVID-19 infection. We then simulated the survival of every individual in the population with a positive COVID-19 test, with and without the assumption that COVID-19 affected their survival, taking account of their personal vulnerability. We used the difference between these simulated survival times to estimate mortality displacement (how long those who died would have lived, had they not tested positive). We used the displacement estimates for those aged 65 and older to revise estimates of excess deaths.

**Results:**

We estimated median mortality displacement of 4.8 (IQR = 1.5 to 16) years for females and 4.4 (IQR = 1.4 to 12.6) years for males at ages 65 and over. We estimate 28% of those dying with COVID-19 aged 65 and over would have survived five years or more without the infection (66% for females aged 65–74).

**Conclusions:**

Life expectancy of those who died with COVID-19 was substantial and, based on our analysis of vulnerability, most of those who died at ages 65 and over are unlikely to have been close to death. In future pandemics, real-time modelling of displacement would be helpful in assessing the mortality impact of the pandemic.

## Introduction

The SARS-CoV-2 (COVID-19) pandemic caused prolonged periods of excess mortality across many countries globally [1–3]. Many individuals that died with COVID-19 infection died earlier than they would have done without the disease – so-called ‘mortality displacement’ [4]. Critics of the pandemic response have suggested that those that died were mostly very vulnerable and close to death, i.e., with minimal displacement [5–7]. If this were true, the economic impact of measures to control the pandemic could not be justified in terms of any benefits in controlling mortality. However, it is statistically complex to estimate overall years of life lost due to COVID-19 infection, particularly in elderly populations, and the magnitude of mortality displacement, as this cannot be undertaken using observed data without undertaking a long-term retrospective analysis comparing mortality for people who lived after contracting COVID-19 infection with those whose risks were similar in other key respects. As such the results a long term study would be of purely historic interest and would not inform preparedness for possible future pandemics.

Quantifying the extent to which deaths have been displaced by COVID-19 infection is also crucial when estimating subsequent expected mortality rates for excess mortality statistics. If individuals with COVID-19 died earlier than they would otherwise have done, this reduces the number of deaths expected in the future. Adjustments should therefore be made to future expected numbers to take account of timing of these COVID-19 related deaths to avoid overestimating future expected mortality and therefore underestimating any observed excess mortality.

To date, estimates of displacement for shock events such as heat waves and influenza have been drawn from aggregate model-based approaches such as Poisson or non-linear time lag models, or by simply comparing excess deaths and deficits in successive time periods [8–11]. However, these approaches do not take into account an individual’s risk of mortality prior to infection, nor can they take into account individual risk factors such as vaccination status and wave of infection [12–16]. Aggregate displacement models also consider the whole population is ‘at risk’, but for the COVID-19 pandemic the risk of death being brought forward is only markedly increased once an individual has contracted COVID-19 [15].

Mortality displacement due to the COVID-19 pandemic has been reported in previous studies, but not quantified as a result of the COVID-19 infection, nor used to adjust estimates of expected mortality over the pandemic period [4,11,17]. Several studies aiming to quantify life lost due to COVID-19 have attempted to adjust estimates by accounting for expected comorbidities in deaths due to other long-term conditions, however these did not fully adjust for underlying health status of individuals and used external estimates of comorbidity not directly linked to the population of interest [18,19]. A study of the Ligurian population in Italy used a Poisson distributed time series approach to model excess mortality and estimated short term mortality displacement of 24% during the local peak of the pandemic. However, the method could not quantify the time lost or survival deficit due to COVID-19 [20].

This study attempts to address the knowledge gap in mortality displacement in the presence of COVID-19 infection across the England population, rather than recorded COVID-19 mortality which will have certification bias in its coding. It uses person-level risk information in a complete England population register to assess the impact of COVID-19 in terms of overall years of life lost and survival deficit. It uses the results of a Cox regression analysis to simulate potential survival times for all known people with a positive SARS-CoV-2 test result in England comparing scenarios in which the person did or did not have COVID-19. Information used on each person’s individual risks include demographic and geographic status, comorbidities, clinical vulnerability and vaccination status [21]. Using this method with a complete population register of England patients we 1) summarise the distribution of life lost with COVID-19 using mortality displacement, and 2) adjust expected mortality based on displacement experienced by groups of individuals with similar levels of risk.

## Methods

### Study design and data sources

We conducted a linked, retrospective cohort analysis of the population of England, based on all individuals registered with the NHS, followed up from 1 March 2020 until 2 September 2022. For the 62,564,742 registered individuals who were born before the 1 March 2020 and registered as alive, 0.3% (n = 185,886) of individuals were excluded due to missing age or sex. We also excluded those who had a SARS-CoV-2 test prior to 1 March 2020, SARS-CoV-2 tests conducted post-mortem, and records where a vaccination date was prior to 1 March 2020 (n = 4593, < 0.01%). All individuals who had a positive COVID-19 test occurring after 31 March 2022 were censored at the week prior to a positive test, which was the point after which widespread community testing was stopped. The following data were linked to the records of these individuals: their Hospital Episode Statistics (HES) records, Office for National Statistics (ONS) mortality record, COVID-19 vaccination and positive SARS-CoV-2 test results. Full details of the study cohort, exclusions, data sources, data fields and linkage used in this stage of the analysis have been published previously [21]. In summary, study data was accessed from 23 November 2022 and identifiers were removed prior to researcher access and analysis.

### Statistical analysis

We used a Cox Proportional Hazard model, with a calendar time baseline hazard function to calculate the hazard ratio related to a positive SARS-CoV-2 test adjusted for risk factors, including sociodemographic, clinical, and health characteristics for each 5-year age group and sex strata. The Primary outcome used was all-cause mortality risk for people within the cohort. The Cox model provided the components to derive the survival profile of individuals and show the temporal pattern of increased risk associated with COVID-19 infection.

We identified all those with positive COVID-19 test results in the period up to 31 March 2022 and followed up their mortality (from any cause of death) from the date of their first positive test until 2 September 2022. Due to legal restrictions on use of the data after the pandemic, mortality was not followed up post September 2022. We used the Cox regression hazard ratios with time-dependent covariates to estimate the probability distribution of the displacement of mortality over time, derived from the risk of dying from any cause after a first positive test result.

We previously described this methodology in Holleyman et al (2023) [15]. It comprised (1) estimation of individuals’ risk of dying, following a first positive test, based on their individual risk factors, and (2) simulating a distribution of survival times assuming no positive test, but using all the other risk factors.

### Risk factors for death

We adapted our earlier Cox regression model of the risk of dying from COVID-19, to optimise prediction of the simulation of survival risks [21]. Models to estimate individuals’ risk of dying were fitted in age (five-year) and sex (male/female) groups. We included the following socio-demographic risk factors for individuals: ethnicity, social deprivation, region of residence and care home status. Health characteristics, smoking status and comorbidities were derived using disease specific International Classification of Diseases 10 (ICD-10) and Systemized Nomenclature of Medicine (SNOMED) codes identified from Hospital Episode Statistics (HES) and GP Extraction Services (GPES) records respectively [22,23]. HES diagnoses were taken from March 2015 to February 2020 and GPES diagnoses from any patient record held on GPES prior to 11th November 2021. Health characteristics and comorbidities included: history of asthma, atrial fibrillation, cancer, heart failure, palliative care, cardiovascular disease, diabetes, chronic kidney disease, dementia, coronary heart disease, learning difficulties, stroke and transient ischaemic attack, liver cirrhosis, epilepsy, bipolar disorder and schizophrenia. In addition to the major health conditions, additional comorbidities were selected on the basis of previous research suggesting an increased risk of mortality from COVID-19 and conditions that have a relatively high prevalence and a more immediate impact on mortality in order to capture the risk on death rates within a relatively short follow-up period [24]. Hypertension was excluded as the clinical recording of hypertension appeared to decrease the hazard ratio and therefore appeared protective. Obesity was excluded due its association with other health conditions and likely a collinear effect. We tested the proportional hazards assumption in the 70–74 age group for all fixed time covariates by plotting Schoenfeld residuals over time. No clear trends over time were evident for any of the covariates.

Time dependent effects based on the first COVID-19 vaccination status and 3 waves of COVID infection were also included. Waves were defined by the times at which pandemic levels increased and decreased. Further information on the specification of the model, the covariates, and wave definitions have been published previously [21].

### Simulating distributions of survival: mortality displacement

We used the hazard ratio coefficients obtained from the Cox regression model in order to simulate survival for each individual aged 65 or older in the cohort who tested positive for COVID-19. For each individual 1,000 simulations of mortality outcome by week were performed to predict their cumulative survival probability from March 2020 to September 2022. The simulation process firstly calculated survival outcome (death or survival) or censoring for each person for each week period over 130 weeks of follow-up using the combined hazard derived from the Cox regression coefficients and derived baseline hazard. Secondly, the outcomes at each time point were transformed into a survival curve from the start of follow-up to death or the end of follow-up time at which point they were censored. This was repeated 1,000 times, providing survival times for each person, including censoring status if the patient survived to the end of the follow-up period. Median displacement was calculated for every simulated individual under two different simulated scenarios:

1. the ‘COVID-19 infection scenario’, where the full range of risk factors were included and;
2. the ‘no-COVID-19 infection scenario’, where we assume no contribution of COVID-19 to death by setting the time-dependent COVID-19 positive test coefficients to zero.

The difference between these individuals’ COVID and no-COVID median survival times were summarised as a probability distribution of estimated mortality displacement, by age, sex and wave of infection. The median of the distribution of all individuals was used as a central estimate of years of mortality displacement due to COVID-19 infection in the population. The simulation process, described above, gave 2,000 probability calculations of mortality outcome for each of 1,329,920 million individuals aged 65 or older for up to 130 time points per individual, with the overall results summarised for each of those 12 age/sex strata by the medians of the individual level survival medians. This represented the maximum feasible simulation scale given available computational resources. Further details of the simulation process are provided in S1 Appendix.

### Expected survival and excess mortality adjustment

We extracted from the data published by the Office for Health Improvement and Disparities (OHID), counts of deaths by week with a mention of COVID-19 on the death certificate in the period 23 March 2020 to 30 December 2022 [25]. This time period was extended beyond that used in the displacement analysis stage to allow for the duration of survival among those who died following an initial positive test for COVID-19.

By applying summarised simulated probability distributions to the total deaths, by age and sex, we estimated survival probabilities had they not had COVID-19 at 1 year or less, 1–3 years, 3–5 years, or 5 years and more.

We then used a heuristic approach, based on the empirical distribution of the simulated mortality displacement, by each age-group and sex stratum, to redistribute the weekly deaths with a mention of COVID-19 to the week in which they would have occurred in the absence of COVID-19. Each distribution provides the time adjustment for COVID-19 deaths within that stratum for any week in time where a death was reported. For each week *t* when deaths where a cause of COVID-19 occurred, the total deaths were re-distributed in time according to the probabilities for each week of the strata survival distribution, before being removed from the corresponding strata of expected deaths for weeks *t + 1*, …, *t + k*, with k representing the final week of expected mortality available to the current study. We described this approach with mathematical notation in Holleyman et al, 2023 [15].

Using these weekly figures, we adjusted the expected numbers of deaths in each week from the published mortality point estimate data from OHID Excess Mortality Reports [25], to derive adjusted estimates of excess mortality had the pandemic not occurred (by sex and the three 10-year age groups 65–74, 75–84, and 85 and over) in England for period 23 March 2020 to 30 December 2022. Further details are provided in S1 Appendix.

### Study approvals

Public Health England (PHE) had legal permission, provided by Regulation 3 of The Health Service (Control of Patient Information, or COPI) Regulations 2002, to process confidential patient information without consent under Sections 3(i) (a) to (c), 3(i)(d) (i) and (ii) and 3(3) as part of its responsibility to manage the COVID-19 pandemic response [26]. As such, this work was carried out as part of Public Health England and the Department of Health and Social Care’s responsibility to monitor the health of the population, under pandemic regulations within the COPI regulation period to June 2022 and did not require ethical approval. Use of the identifiable datasets for this project was approved by (1) Caldicott Guardians for the UK Health Security Agency (UKHSA) and the Department of Health and Social Care (DHSC), (2) the DHSC Data Protection Officer and Senior Information Risk Owner, when those organisations took over responsibilities from Public Health England (PHE). Further information is provided in S1 Appendix.

## Results

Details of our dataset have been published previously [21]. In summary, 62,564,742 individuals registered with the NHS in England were born before 1 March 2020 and recorded as alive on that date. Of these, records of 62,374,261 individuals were included in our analysis (99.7%). Of those included in the analysis, 15,918,831 (25.5%) tested positive for COVID-19 between 1 March 2020 and 31 March 2022. Table 1 summarises the characteristics and vital statuses of individuals included in our analysis. The full demographic and health characteristics of the population are presented in S1 Table.

**Table 1:** COVID-19 positive test status and first vaccination status by mortality outcome in the period 1 March 2020 to 31 March 2022, for England cohort (n = 62,374,261).

Variable	Level	Alive (%)	Died (%)	Total
Total N (%)		61,017,385 (97.8)	1,356,876 (2.2)	62,374,261
COVID-19 positive test status by wave	No COVID	45,372,384 (97.7)	1,083,046 (2.3)	46,455,430
	Wave 1	281,788 (81.9)	62,154 (18.1)	343,942
	Wave 2	2,983,916 (95.8)	131,854 (4.2)	3,115,770
	Wave 3	12,379,297 (99.4)	79,822 (0.6)	12,459,119
1st COVID-19 vaccine	Not Vaccinated	16,841,894 (96.3)	642,356 (3.7)	17,484,250
	Vaccinated	44,175,491 (98.4)	714,520 (1.6)	44,890,011

### Risk factors for death

The Cox regression analysis showed that SARS-CoV-2 positive test increased the risk of death across all modelled age groups. This elevated risk generally reduced with time after the positive test and over waves of the pandemic. Having a COVID-19 vaccination was associated with a reduction in risk of death after testing positive. These results are similar to our earlier paper and full details and interpretation of this modelling can be found elsewhere [21]. The full list of relative risks for modelled covariates by age group, for those aged 35 and over, are included in S2 Table.

### Distribution of mortality displacement

Survival times by week were simulated for 2,785,918 individuals aged 55 years or older who tested positive for COVID-19 between 1 March 2020 and 31 March 2022. Simulations on subjects aged 55–64 who had a COVID-19 positive test on 28 March 2021 or later had a median of zero weeks mortality displacement, with a lower 25th centile of the distribution indicating a negative displacement. We assumed this reflected a poor predictive effect for a number of reasons (see simulation process in S1 Appendix for a discussion of this) and this would apply to younger ages as well. For this reason, only simulated displacement for individuals aged 65 years or older (n = 1,329,920) were assumed to be valid with the current study methodology. The distribution of mortality displacement by age/sex group is presented in S1 Fig.

**Fig 1:**
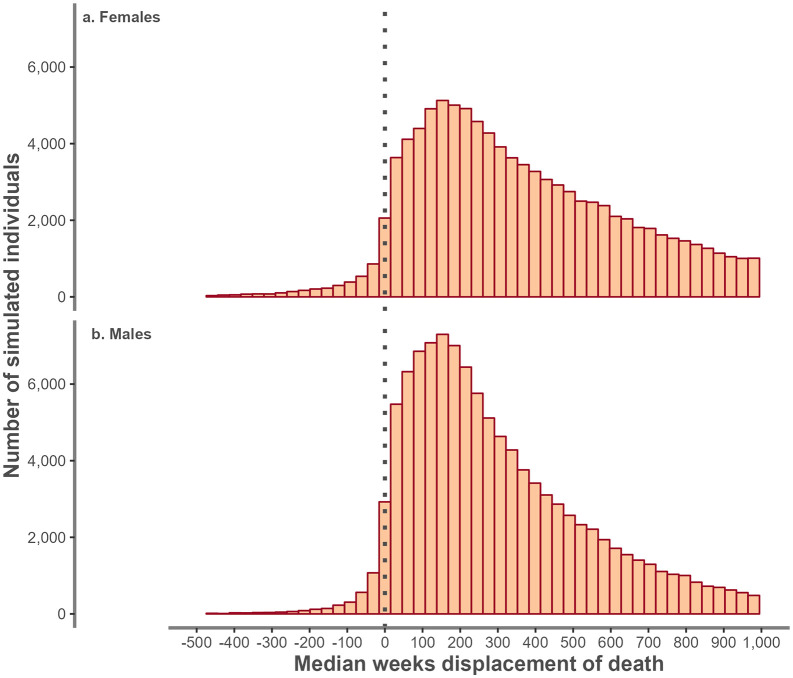
Distribution of the number of weeks of displacement of time of death among individuals that tested positive for COVID-19 in England by sex among individuals aged 75 to 79 years.

For illustration, Fig 1 presents, for individuals aged 75–79 years, the number of weeks each person in England who died following a positive test for COVID-19 would have been expected to live, had they not tested positive for COVID-19. This is displayed as a distribution of individuals’ reported weeks displacement, separately for females and males. The median displacement in this age group was 351 (IQR 164–657) and 247 (IQR 122–455) weeks for females and males respectively (S3 Table).

**Fig 2:**
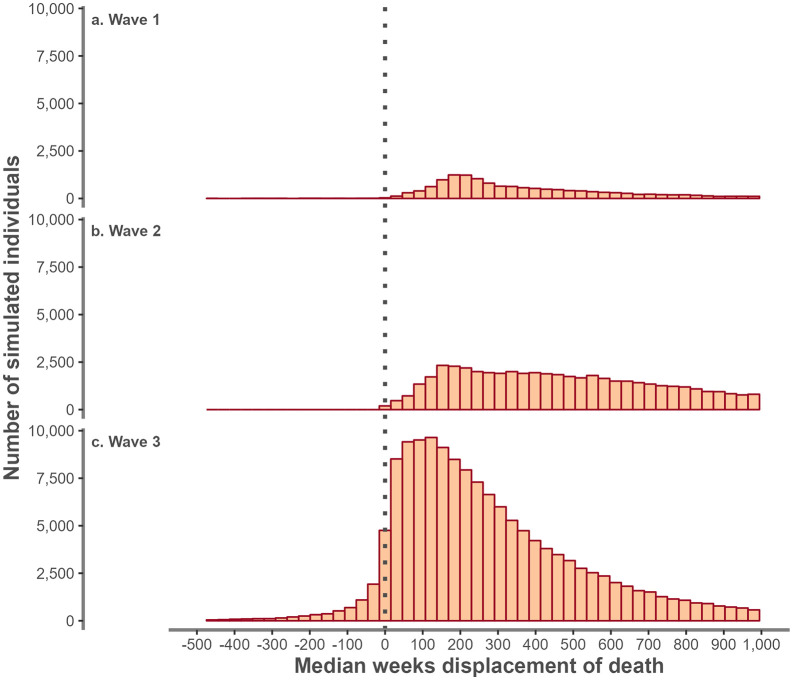
Distribution of the number of weeks of displacement of time of death among individuals that tested positive for COVID-19 in England by wave of positive test among individuals aged 75 to 79 years.

The distribution of displacement differed by the wave in which an individual’s first positive test occurred. Figure 2 illustrates the distribution of displacement by wave for individuals aged 75–79 years.

For females aged 65 or older, testing positive for COVID-19 for the first time between 28 September 2020 and 27 March 2021 (wave two) showed greater displacement compared with other waves, with a median of 448 weeks (IQR 146–1,323). In this age/sex group, displacement was least between 1 March 2020 and 27 September 2020 (wave one) with a median length of 164 weeks (IQR 508–449).

For males aged 65 or older, the greatest amount of displacement was also seen in wave two (median: 486 weeks, IQR: 854–1,172) compared with the least in the period 28 March 2021 to 31 March 2022 (wave three) with a median of 173 weeks (IQR 342–491). S1 Fig presents the distribution of displacement by sex and age groups 65 or older and these are summarised in S3 Table. A summary of the distributions by age group and wave are summarised in S4 Table. In all age groups median displacement increased in wave two compared to wave one with the smallest displacement estimated in wave three. S4 Table indicates the widest range of displacement was found in the 65–69 age group with negative displacement in at least 25% of the individuals in wave three.

Table 2 presents summary median years (converted from weeks) of mortality displacement of individuals in England who tested positive for COVID-19 by sex and age group, based on their simulated survival times. For all ages 65 + , the median years of displacement was 4.8 years (IQR = 1.5 to 16) for females and 4.4 years for males (IQR = 1.4 to 12.6). The displacement was greatest in the 65–69 age group with a median of 14.4 years (IQR = 0.5 to 38.8) for females and 9.9 years (IQR = 1.1 to 26.2) for males. Displacement decreased with age, being lowest in the 90 and over age group with a median of 1.4 years (IQR = 0.8 to 2) for females and 1.2 years (IQR = 0.6 to 1.7) for males.

**Table 2:** Median years displacement (and interquartile range) of COVID-19 positive subjects in England by sex and age group, 65 years and older (n = 1,329,920).

Age Group	Females	Males
65-69	14.4 (0.5-38.8)	9.9 (1.1-26.2)
70-74	10.6 (3.1-23.3)	7.2 (2.4-15.8)
75-79	6.8 (3.2-12.6)	4.7 (2.3-8.7)
80-84	3.4 (1.9-6.1)	2.6 (1.4-4.4)
85-89	1.9 (1-3.1)	1.6 (0.8-2.5)
90+	1.4 (0.8-2)	1.2 (0.6-1.7)
65+	4.8 (1.5-16)	4.4 (1.4-12.6)

### Expected survival without COVID-19

The proportions of people who tested positive for COVID-19 in England who would otherwise have been expected to survive at least 5 years varied by age and sex – 66% of females and 61% of males at ages 65−74, but 6% and 3%, respectively at ages 85 and over. By applying the summary displacement proportions, by age and sex and duration, for those testing positive for COVID-19, to all deaths with COVID-19 recorded on their death certificates, we summarise how long people aged 65 or older, who died with COVID-19 between 27 March 2020 and 30 December 2022, were expected to live had they not had the disease (S5 Table). We estimate that 23.5% of deaths aged 65 and over would not have been expected to survive more than one year. However, 28% would have been expected to have survived for 5 years or more had they not had the disease.

### Adjusted expected deaths and excess mortality

Over the period 27 March 2020 to 30 December 2022, the unadjusted excess mortality model used by OHID estimated that there were 115,242 deaths at ages 65 years or older in excess of what would have been expected on the basis of trends in mortality in the five years prior to the pandemic. After adjusting the number of expected deaths for mortality displacement by age and sex, using the above survival time distributions, the estimated number of excess deaths over the same period increased by 58,408 to 173,651 (Table 3). This represents an estimate of the total number of people who died earlier than expected as a result of the pandemic. The proportional adjustment was higher for males compared with females, and highest in males aged 85+ (from 21,134 to 42,468).

**Table 3:** Cumulative adjusted and unadjusted excess mortality for England by sex and age group, between 27 March 2020 and 30 December 2022.

Sex	Age group	Unadjusted excess mortality	Adjusted excess mortality
Females	65-74	8,836	9,564
	75-84	17,737	21,989
	85+	28,957	50,963
Males	65-74	15,297	17,199
	75-84	23,282	31,468
	85+	21,134	42,468
Total		115,243	173,651

**Fig 3:**
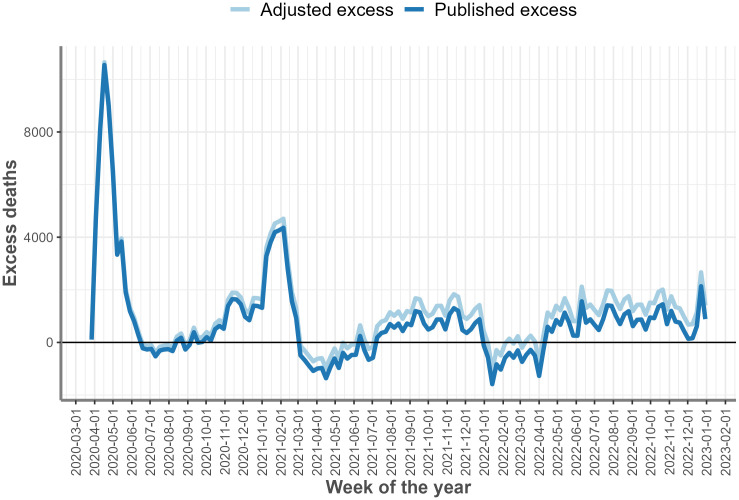
Adjusted and published unadjusted excess deaths for England between 27 March 2020 and 30 December 2022.

The difference between the adjusted and published (unadjusted) estimates increased progressively from 27th March 2020 to 30 December 2022 (Fig 3) but followed the same weekly pattern. For this reason the weeks in which peaks and troughs occurred did not alter.

## Discussion

In this paper, we have (1) quantified the extent of mortality displacement due to COVID-19 infection by age and sex, (2) used these figures to estimate how long people aged 65 or older who died with COVID-19 recorded on their death certificate would have been expected to live had they not had the disease, and (3) estimated the extent to which excess mortality was underestimated progressively. To our knowledge, this is the first study to estimate mortality displacement using data on socio-demographic, health and COVID-19 vaccination status at individual level for a complete national cohort of people aged 65 or older.

Overall, our results for ages 65 or older confirm that the life expectancy of those who died with COVID-19 would otherwise have been substantial even after assessing individual risk factors and existing comorbidities. At ages 65 and older, 28% were expected to live for five years or more (with this figure being 66% and 61% for females and males at ages 65–74). Among those with short expected survival, comorbidities and age appear to be likely to have been significant factors in their deaths. Uncertainty around the median estimated years displacement presented in the paper is provided in the form of inter-quartile ranges. Each individual’s 1,000 simulated survival times are summarised by the median, and the median of these medians for all individuals in each age/sex group is taken and reported. Calculating confidence intervals around these medians of medians would not capture the uncertainty in the individual level medians resulting from the simulations, and they would be very narrow and give a false impression of certainty. The interquartile range gives a fair and easily understandable summary of the uncertainty, given the complexity of the methodology.

There was substantial variation in the estimated displacement of all individuals both by age and sex with high levels of uncertainty in the median estimates, which suggests that although the simulations indicate general displacement in the presence of COVID-19 infection, a single central estimate has considerable uncertainty within these stratified groups. In the present study we also saw a much wider range of predicted displacement from the simulated distributions in wave three compared to earlier waves of the pandemic, indicating a less consistent effect of COVID-19 infection in estimating a higher mortality risk compared to not having COVID-19 infection in the general population. Part of this effect may reflect the reduction in COVID-19 death rates over time in the general population, which reflect factors such as greater rollout of vaccinations and lowered risk associated with later virus variants such as Omicron [27,28].

Correcting for mortality displacement had a large impact on weekly estimates of excess mortality, particularly a year or more into the pandemic. Doing so allows for increased precision in detecting any increases in rates of death from causes such as cancer and cardiovascular disease, which may be impacted by periods of reduced access to screening or healthcare or possible adverse impacts of COVID-19 containment measures such as those related to social isolation.

Our approach has several limitations. We were unable to estimate the impact of mortality displacement at ages less than 65 years due to lack of predictive ability of the model to estimate mortality difference between having and not having a first COVID-19 infection in the presence of all other available covariates in the model. Given that displacement among individuals that died in younger age groups is likely to be many years, using a life table method may be more useful for the general population group, or developing models for specific risk groups using other clinical data sources that may have less generalisability to the wider population [15].

Even for those aged 65–74, the simulation process resulted in estimates, for a substantial number of people, where survival in the absence of COVID-19 was shorter than survival with the disease, resulting in negative displacement outcomes. Producing a larger number of simulations may have reduced this effect, but some of this may be due to the underlying health of the patient, coincidental detection of nosocomial COVID-19 infection in some patients already at the end of life, the predictive ability of the model with the covariates available, and the relatively short follow-up time in the Cox survival model.

We only account for the first positive test for COVID-19 and do not account for the changes in risk as a result of either subsequent infections or conversely a long, subsequent period free of infection. In our model, anyone who did not have a positive test recorded was treated as being free of COVID-19 and are therefore included in the baseline risk. As there were likely to be many people, particularly in wave one who had COVID-19 and died without recording a positive test, this will result in an underestimation of the overall impact of displacement. It is also likely that in wave one, a higher proportion of those tested had more serious existing health problems than the proportion in wave two, which may have accounted for the larger displacement seen in patients in wave two [29,30]. We were also limited to a relatively short follow-up period due to the legal restrictions of the time limited use of the data sources, and most of the deaths we predict to occur do so after our follow-up period.

Our work adds to a body of literature around the impact of the COVID-19 pandemic on mortality. There has been some focus on years of life lost as governments have tried to quantify the impact of the pandemic on mortality rates, particularly with life expectancy in 2020 dropping for women and men respectively relative to 2019 levels [5,31,32]. Existing analyses of the years of life lost to COVID-19 in England suggest that on average, lives have been cut short in 2020 by between 9 and 11.5 years using life table methods [6,7,33,34]. Between 2020 and 2022 the average life expectancy for England was higher than our simulated median mortality displacement due to COVID-19 for all ages from 65 or older [35]. We suggest our lower displacement estimates are a function of using individual survival, which takes into account an individual’s risk of death, rather than relying on a life table method.

Our findings have important implications for public health in England. Measures of the impact of the COVID-19 pandemic on mortality should be adjusted to account for displacement when quantifying this historically, or they will underestimate the effect [6]. We provide evidence that the effect of mortality displacement for people over the age of 65 years varies by age group, sex and wave of infection, though we have not estimated how it varies between other population groups for whom there were disparities in COVID-19 outcomes, such as by ethnic group and region. Further work to estimate this impact among these groups would be needed.

Importantly, our method provides a framework for undertaking mortality impact assessment at the individual level, following a population disruption event, such as a future pandemic.

Overall, the claim that those who died were very close to death is not supported by our analysis.

## Supporting information

S1 Appendix Additional information. (DOCX)

S1 Table Sociodemographic and health characteristics of the population by age groups, gender, and COVID-19 positive test status. (DOCX)

S2 Table Hazard ratios (and 95% confidence intervals) derived from the Cox Proportional Hazard Model denoting the relative change in death rates, stratified by sex and age group (35+). (DOCX)

S3 Table Median weeks (and interquartile range) of distribution for displacement of mortality by age group and sex, 65 years or older, England. (DOCX)

S4 Table Median weeks (and interquartile range) of distribution for displacement of mortality by age group and wave, 65 years or older, England. (DOCX)

S5 Table Estimated percentage (%) of people aged 65 or older who died with COVID-19 by expected length of survival had they not had the disease, by sex, 27 March 2020 to 30 December 2022. (DOCX)

S1 Fig Simulated distribution (weeks) for displacement of mortality by age group and sex, 65 years or older, England. (TIFF)

S2 Fig Simulated distribution (weeks) for displacement of mortality by age group and wave, 65 years or older, England. (TIFF)
