# Supplementary material for: Life lost due to the COVID-19 pandemic: A model-based cohort analysis of mortality displacement in the registered population of England
Source: PLoS One. 2026 May 8;21(5):e0348575. doi: 10.1371/journal.pone.0348575 (PMC13155604; doi:10.1371/journal.pone.0348575)
Supplement: S1 Appendix — (DOCX) [file pone.0348575.s001.docx]

**Appendix S1 – Additional information**

**Data and approvals**

Use of the identifiable datasets for this project was approved by (1) Caldicott Guardians for the UK Health Security Agency (UKHSA) and the Department of Health and Social Care (DHSC), (2) the DHSC Data Protection Officer and Senior Information Risk Owner, when those organisations took over responsibilities from Public Health England (PHE). The weekly excess mortality surveillance process makes use of identifiable data in order to link datasets. Identifiers were removed prior to analysis for this study. Approval to link each of the datasets to each other, for this purpose, was obtained prior to using them from all of the data owners concerned. All approvals are documented and available on request.

**Statistical modelling**

Separate multivariable Cox Proportional Hazard models were fitted for each 5-year age strata to estimate the relative increase or decrease in the risk of death associated with the exposure variables. These included time-fixed and time-dependent terms. A positive SARS-CoV-2 test was modelled as a time-dependent term using dummy variables to allow the effects of COVID-19 to vary by time after infection, wave in the pandemic, and vaccination status. A vaccine was defined as taking effect if it was administered 2 weeks prior to a positive test. Due to the timing of vaccine availability and rollout (from December 2020), vaccine status was modelled in such a way that the effect of vaccine was not applied during wave one. All other covariates were modelled as fixed effects.

We tested the proportional hazards assumption by plotting Schoenfeld residuals over time for a random 50% sample of the cohort for males (n = 684,247) and females (n = 740,444) aged 70 to 74 years, then running a model using all fixed time covariates only. No trends over time were evident in the plots for any of the covariates in the model that reported a statistically significant effect for violating the proportional hazards assumption. We compared convergence in the fixed covariate model with a complete model including time dependent variables with Harrell’s C-statistic. The C-statistic increased from 0.75 (standard error 0.001) in the fixed effects only model compared to 0.78 (standard error 0.001) when including time dependent terms for males. For females the C-statistic increased from 0.78 (standard error 0.001) for fixed terms only to 0.80 (standard error 0.001) when including time dependent terms.

Complete models for younger age groups (0 to 35 years) failed to converge because the numbers of deaths were too small. Further details of the statistical modelling have been published previously Bauer-Staub et al.^1^

**Simulation process**

For each individual that tested positive for COVID-19, 1,000 simulations of mortality outcome were performed. Simulations were carried out iteratively for the oldest 5-year age-group descending until results showed no displacement. Individuals’ combined hazard was estimated from the model terms extracted from the statistical model in order to predict their cumulative survival probability under two different scenarios:

1. the ‘COVID-19 infection scenario’, where the full combined hazard is included and
2. the ‘no-COVID-19 infection scenario’, where we assume no contribution of COVID-19 to death by setting the time-dependent COVID-19 positive test coefficients (the log hazard) to zero.

Simulations were performed for each individual for the COVID-19 scenario and no-COVID-19 scenario until either death or until the maximum follow-up time defined by the model (130 weeks) was reached. In order to estimate the median date of death under either scenario, the 1,000 simulated dates of death (and censoring statuses to incorporate those that survive through all failure times) in each individual were treated as if they were survival times in a study population and summarised using a conventional Kaplan-Meier plot.^2^ If the Kaplan-Meier survival function in each subject fell below 50%, the median was estimated as the particular survival time at which the survival function first dropped below 50%. If the survival function never fell as low as 50% no direct estimate existed for the median survival time, so the survival curve was first linearised by taking the natural logarithm of the survival probabilities. The total curve was extrapolated based on the best fitting straight line and the median survival identified as the survival time at which that straight line fell below 50%.

The difference between the median survival time for the COVID-19 and no-COVID-19 scenarios for each individual provided the average mortality displacement for that individual. Further details on methods of simulation are outlined in the in Holleyman et al (2023).^3^

Survival times were simulated for each 5-year age group and sex starting at the oldest (90 years or older) descending. We reviewed the distributions of (both median and percentiles) mortality displacement time results by age and sex. Where simulated mortality displacement had at least 25 percent of people with a negative displacement and the median displacement zero we removed the age-group from the final analysis, under the assumption of poor predictive effect. We assume a poor predictive effect for a number of reasons including 1) COVID-19 coefficients having limited effect on predicting mortality survival time; 2) limited deaths (due to the lack of follow-up time available) in the underlying age group provides too few ‘failures’ in the Kaplan-Meier analysis, which in turn providing too many random outcomes or 3) Simulation numbers were not large enough to ensure a robust median survival. We simulated 2,785,918 individuals aged 55 years or older who tested positive for COVID-19 between 1 March 2020 to the 31st March 2022, but only ages 65 or older were included in the final analysis.

**Excess mortality displacement adjustment**

Data for estimates of national weekly expected mortality, excess mortality and observed COVID-19 mortality (based on death certificate records) were obtained from the Office of Health Improvement and Disparities (OHID) weekly excess death reports).^4^ Data was available by sex and age group and data was selected for people aged 65 or older for three 10-year age groups (65-74, 75-84 and 85+ years). The median difference survival times for each individual aged 65 or older in the simulation dataset were summarised into distributions of mortality displacement matching sex and age group those available from the OHID mortality data. Distributions were created using a kernel density function for each group. Using these distributions by each sub-group, we applied a ‘ball and urn approach’ to adjust published excess mortality estimates for displacement from the week ending 27th March 2020 to the week ending 30th December 2022.^3^

For the ‘ball and urn’ approach, three sets of metrics were required: 1. the empirical distribution of mortality displacement generated through the simulations; 2. COVID-19 deaths at a given time $t$; and 3. estimates of expected deaths at $t$ from the OHID excess deaths model.^13^ For this step we adopted a heuristic approach to adjusting the expected number of deaths in any given week, based on the difference in expected survival time under COVID and no-COVID scenarios $\Delta_{j}$ - applied to people who died from COVID in week $t$. The analysis is perhaps best illustrated via a practical simplified example based on a “ball and urn” model as outlined in Figure A.

In the simplified example illustrated in Figure A, the empirical distribution of $\Delta_{j}$ falls between one and six weeks, with no negative values of $\Delta_{j}$ being observed. In fact, 10% of the deaths are positively displaced by one week, 20% by two weeks, 30% by three weeks, 20% by four weeks, 10% by 5 weeks and 10% by six weeks. Using this empirical distribution, the expected deaths in weeks $t$+1,…, $t$+6 are adjusted by subtracting the corresponding number of COVID deaths from week $t$, resulting in an adjusted expected deaths estimate. This process is carried out iteratively, week by week, on each occasion removing the appropriate number of expected deaths from the down-stream weeks where they would have been expected to fall in the absence of COVID-19 deaths. Further details of this approach, including some of the assumptions are provided in Holleyman et al (2023).^3^


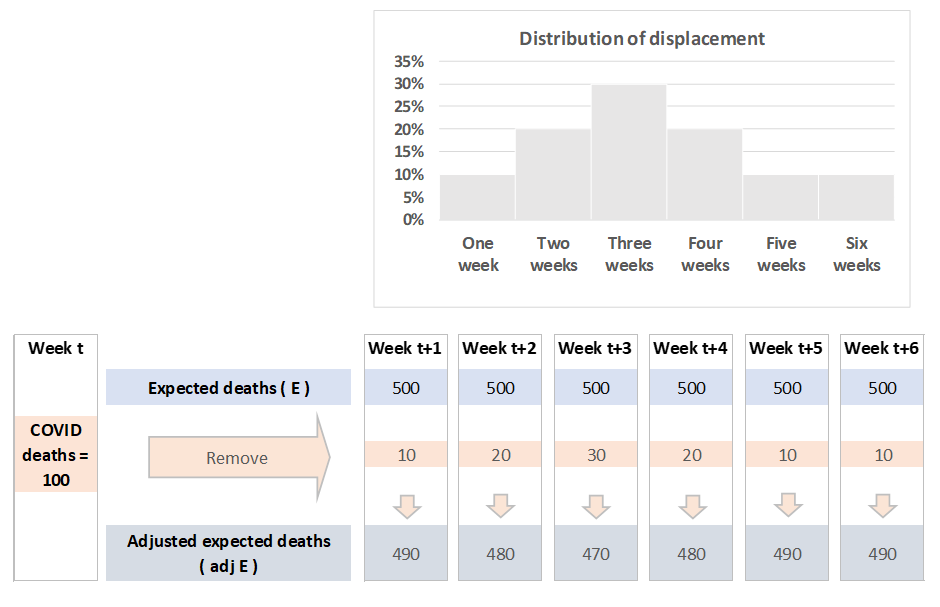


*Figure A – Simple example for adjusting expected deaths using displaced COVID-19 deaths.*

**Technical glossary**

Cox Regression: Cox regression (or proportional hazards regression) is method for investigating the effect of several variables upon the time a specified event takes to happen. In the context of an outcome such as death (in the current study) this is known as Cox regression for survival analysis.

Hazard ratio (HR): A measure of how often a particular event happens in one group compared to how often it happens in another group, over time. It compares the rate (or "hazard") of an event (like death, disease, or recovery) occurring in an experimental group versus a control group over time, calculated as the ratio of their instantaneous risks (HR = Hazard_treated / Hazard_control).

Interquartile range: It is defined as the difference between the 75th and 25th percentiles of the data. In the current study the 25^th^ and 75^th^ percentile points are reported.

**References**

1 Bauer-Staeb C, Holleyman RJ, Barnard S, Hughes A, Dunn S, Fox S, et al. Risk of death in England following a positive SARS-CoV-2 test: A retrospective national cohort study (March 2020 to September 2022). PLOS One. 2024; 19: e0304110. doi: 10.1371/journal.pone.0304110. PMID: 39383163; PMCID: PMC11463829.

2 Cox DR· Regression Models and Life-Tables· *Journal of the Royal Statistical Society: Series B (Methodological)* 1972; **34**: 187–202·

3 Holleyman RJ, Barnard S, Bauer-Staeb C, Hughes A, Dunn S, Fox S, et al. Adjusting expected deaths for mortality displacement during the COVID-19 pandemic: a model based counterfactual approach at the level of individuals. BMC Medical Research Methodology. 2023; 23: 241. doi: 10.1186/s12874-023-01984-8. PMID: 37853353; PMCID: PMC10585864

4 Office for Health Improvement and Disparities· Excess mortality in England and English regions· GOV·UK· 2023; published online March 9· https://www·gov·uk/government/statistics/excess-mortality-in-england-and-english-regions (accessed March 31, 2022)·
