# Supplementary material for: Life lost due to the COVID-19 pandemic: A model-based cohort analysis of mortality displacement in the registered population of England
Source: PLoS One. 2026 May 8;21(5):e0348575. doi: 10.1371/journal.pone.0348575 (PMC13155604; doi:10.1371/journal.pone.0348575)
Supplement: S1 Table — (DOCX) [file pone.0348575.s002.docx]

**Table S1 – Sociodemographic and health characteristics of the population by age groups, gender, and COVID-19 positive test status**

| **Variable** | **Level** | **Alive** | **Died** | **Total** |
| --- | --- | --- | --- | --- |
| Sex | Male | 30,598,886 (97·8) | 685,230 (2·2) | 31,284,116 |
|  | Female | 30,418,499 (97·8) | 671,646 (2·2) | 31,090,145 |
| Age Group | <1 | 623,923 (99·9) | 387 (0·1) | 624,310 |
|  | 01-04 | 2,718,284 (100·0) | 482 (0·0) | 2,718,766 |
|  | 05-09 | 3,648,285 (100·0) | 469 (0·0) | 3,648,754 |
|  | 10-14 | 3,589,312 (100·0) | 683 (0·0) | 3,589,995 |
|  | 15-19 | 3,449,145 (100·0) | 1,696 (0·0) | 3,450,841 |
|  | 20-24 | 4,140,169 (99·9) | 2,441 (0·1) | 4,142,610 |
|  | 25-29 | 4,591,869 (99·9) | 3,771 (0·1) | 4,595,640 |
|  | 30-34 | 4,747,710 (99·9) | 5,798 (0·1) | 4,753,508 |
|  | 35-39 | 4,482,769 (99·8) | 9,082 (0·2) | 4,491,851 |
|  | 40-44 | 4,005,282 (99·7) | 13,030 (0·3) | 4,018,312 |
|  | 45-49 | 4,087,521 (99·5) | 22,419 (0·5) | 4,109,940 |
|  | 50-54 | 4,201,328 (99·2) | 35,673 (0·8) | 4,237,001 |
|  | 55-59 | 3,949,846 (98·7) | 52,049 (1·3) | 4,001,895 |
|  | 60-64 | 3,271,265 (97·9) | 69,263 (2·1) | 3,340,528 |
|  | 65-69 | 2,789,596 (96·7) | 95,024 (3·3) | 2,884,620 |
|  | 70-74 | 2,700,648 (94·8) | 148,735 (5·2) | 2,849,383 |
|  | 75-79 | 1,848,158 (91·0) | 183,323 (9·0) | 2,031,481 |
|  | 80-84 | 1,241,715 (84·3) | 232,093 (15·7) | 1,473,808 |
|  | 85-89 | 653,843 (73·1) | 240,827 (26·9) | 894,670 |
|  | 90+ | 276,717 (53·6) | 239,631 (46·4) | 516,348 |
| Ethnic origin | White | 45,654,247 (97·4) | 1,226,281 (2·6) | 46,880,528 |
|  | Asian | 5,766,985 (99·2) | 46,228 (0·8) | 5,813,213 |
|  | Black | 2,442,030 (99·0) | 24,466 (1·0) | 2,466,496 |
|  | Mixed | 1,245,406 (99·6) | 5,409 (0·4) | 1,250,815 |
|  | Other | 1,207,229 (99·6) | 4,689 (0·4) | 1,211,918 |
|  | Unknown | 4,701,488 (99·0) | 49,803 (1·0) | 4,751,291 |
| Quinitle of  multiple deprivation | Q5 (least deprived) | 11,436,133 (97·9) | 247,099 (2·1) | 11,683,232 |
|  | Q2 | 11,717,733 (97·8) | 266,592 (2·2) | 11,984,325 |
|  | Q3 | 12,086,946 (97·8) | 272,202 (2·2) | 12,359,148 |
|  | Q4 | 12,511,881 (97·9) | 265,027 (2·1) | 12,776,908 |
|  | Q5 (Most deprived) | 12,394,071 (97·8) | 275,307 (2·2) | 12,669,378 |
|  | (Missing) | 870,621 (96·6) | 30,649 (3·4) | 901,270 |
| Region | East Midlands | 5,065,152 (97·7) | 121,457 (2·3) | 5,186,609 |
|  | East of England | 6,925,626 (97·8) | 156,811 (2·2) | 7,082,437 |
|  | London | 10,308,801 (98·7) | 134,099 (1·3) | 10,442,900 |
|  | North East | 2,752,316 (97·4) | 73,983 (2·6) | 2,826,299 |
|  | North West | 7,783,693 (97·6) | 191,751 (2·4) | 7,975,444 |
|  | South East | 9,411,716 (97·8) | 212,054 (2·2) | 9,623,770 |
|  | South West | 5,901,565 (97·6) | 147,841 (2·4) | 6,049,406 |
|  | West Midlands | 6,252,953 (97·7) | 149,556 (2·3) | 6,402,509 |
|  | Yorkshire and Humber | 5,744,942 (97·6) | 138,675 (2·4) | 5,883,617 |
| Smoking status | Never smoker | 24,787,827 (97·9) | 522,832 (2·1) | 25,310,659 |
|  | Current smoker | 6,660,865 (97·7) | 155,190 (2·3) | 6,816,055 |
|  | Ex-smoker | 9,361,759 (95·5) | 441,618 (4·5) | 9,803,377 |
|  | Non smoker | 1,606,533 (97·8) | 35,453 (2·2) | 1,641,986 |
|  | No record | 18,600,401 (98·9) | 201,783 (1·1) | 18,802,184 |
| Clinically vulnerable (NIMS) | No | 57,508,287 (98·5) | 897,348 (1·5) | 58,405,635 |
|  | Yes | 3,509,098 (88·4) | 459,528 (11·6) | 3,968,626 |
| Care Home | No | 60,925,768 (98·0) | 1,251,708 (2·0) | 62,177,476 |
|  | Yes | 91,617 (46·6) | 105,168 (53·4) | 196,785 |
| Wave | No COVID | 45,372,384 (97·7) | 1,083,046 (2·3) | 46,455,430 |
|  | Wave 1 | 281,788 (81·9) | 62,154 (18·1) | 343,942 |
|  | Wave 2 | 2,983,916 (95·8) | 131,854 (4·2) | 3,115,770 |
|  | Wave 3 | 12,379,297 (99·4) | 79,822 (0·6) | 12,459,119 |
| COVID-19 infection | No COVID positive test | 45,372,384 (97·7) | 1,083,046 (2·3) | 46,455,430 |
|  | No record of COVID positive test | 15,645,001 (98·3) | 273,830 (1·7) | 15,918,831 |
| 1st COVID-19 vaccine | Not Vaccinated | 16,841,894 (96·3) | 642,356 (3·7) | 17,484,250 |
|  | Vaccinated | 44,175,491 (98·4) | 714,520 (1·6) | 44,890,011 |
| Conditions |  |  |  |  |
| Asthma (QOF) | No | 57,617,058 (94·4) | 1,248,891 (92·0) | 58,865,949 |
|  | Yes | 3,400,327 (5·6) | 107,985 (8·0) | 3,508,312 |
| Atrial fibrillation (QOF) | No | 60,071,226 (98·4) | 1,128,954 (83·2) | 61,200,180 |
|  | Yes | 946,159 (1·6) | 227,922 (16·8) | 1,174,081 |
| Cancer (QOF) | No | 59,322,384 (97·2) | 1,098,054 (80·9) | 60,420,438 |
|  | Yes | 1,695,001 (2·8) | 258,822 (19·1) | 1,953,823 |
| COPD (QOF) | No | 60,030,333 (98·4) | 1,175,070 (86·6) | 61,205,403 |
|  | Yes | 987,052 (1·6) | 181,806 (13·4) | 1,168,858 |
| Heart failure (QOF) | No | 60,625,293 (99·4) | 1,217,807 (89·8) | 61,843,100 |
|  | Yes | 392,092 (0·6) | 139,069 (10·2) | 531,161 |
| Palliative care (QOF) | No | 60,889,163 (99·8) | 1,218,191 (89·8) | 62,107,354 |
|  | Yes | 128,222 (0·2) | 138,685 (10·2) | 266,907 |
| Cardiovascular disease (HES) | No | 54,877,687 (89·9) | 502,594 (37·0) | 55,380,281 |
|  | Yes | 6,139,698 (10·1) | 854,282 (63·0) | 6,993,980 |
| Chronic respiratory disease (HES) | No | 58,010,646 (95·1) | 1,055,791 (77·8) | 59,066,437 |
|  | Yes | 3,006,739 (4·9) | 301,085 (22·2) | 3,307,824 |
| Diabetes (QOF only) | No | 59,580,926 (97·6) | 1,292,110 (95·2) | 60,873,036 |
|  | Yes | 1,436,459 (2·4) | 64,766 (4·8) | 1,501,225 |
| Diabetes (HES) | No | 59,315,990 (97·2) | 1,087,205 (80·1) | 60,403,195 |
|  | Yes | 1,701,395 (2·8) | 269,671 (19·9) | 1,971,066 |
| Chronic kidney disease (QOF only) | No | 59,970,207 (98·3) | 1,200,980 (88·5) | 61,171,187 |
|  | Yes | 1,047,178 (1·7) | 155,896 (11·5) | 1,203,074 |
| Chronic kidney disease (HES) | No | 60,415,982 (99·0) | 1,126,763 (83·0) | 61,542,745 |
|  | Yes | 601,403 (1·0) | 230,113 (17·0) | 831,516 |
| Dementia (HES or QOF) | No | 60,743,784 (99·6) | 1,111,088 (81·9) | 61,854,872 |
|  | Yes | 273,601 (0·4) | 245,788 (18·1) | 519,389 |
| Coronary heart disease (GPES) | No | 59,489,015 (97·5) | 1,107,596 (81·6) | 60,596,611 |
|  | Yes | 1,528,370 (2·5) | 249,280 (18·4) | 1,777,650 |
| Learning dis· & Downs (GPES) | No | 60,347,053 (98·9) | 1,346,000 (99·2) | 61,693,053 |
|  | Yes | 670,332 (1·1) | 10,876 (0·8) | 681,208 |
| Stroke & TIA (GPES) | No | 60,187,910 (98·6) | 1,180,873 (87·0) | 61,368,783 |
|  | Yes | 829,475 (1·4) | 176,003 (13·0) | 1,005,478 |
| Liver cirrhosis (GPES) | No | 60,933,769 (99·9) | 1,339,158 (98·7) | 62,272,927 |
|  | Yes | 83,616 (0·1) | 17,718 (1·3) | 101,334 |
| Epilepsy (GPES) | No | 60,365,448 (98·9) | 1,322,657 (97·5) | 61,688,105 |
|  | Yes | 651,937 (1·1) | 34,219 (2·5) | 686,156 |
| Bipolar & schizophrenia (GPES) | No | 60,658,155 (99·4) | 1,335,390 (98·4) | 61,993,545 |
|  | Yes | 359,230 (0·6) | 21,486 (1·6) | 380,716 |
