# Supplementary material for: Life lost due to the COVID-19 pandemic: A model-based cohort analysis of mortality displacement in the registered population of England
Source: PLoS One. 2026 May 8;21(5):e0348575. doi: 10.1371/journal.pone.0348575 (PMC13155604; doi:10.1371/journal.pone.0348575)
Supplement: S2 Table — (DOCX) [file pone.0348575.s003.docx]

**Table S2 – Hazard ratios (and 95% confidence intervals) derived from the Cox Proportional Hazard Model denoting the relative change in death rates, stratified by sex and age group (35+)**

|  |  | **35-39 years** | |  | **40-44 years** | |  | **45-49 years** | |
| --- | --- | --- | --- | --- | --- | --- | --- | --- | --- |
|  |  | **Female** | **Male** |  | **Female** | **Male** |  | **Female** | **Male** |
| **Age** | | 1.07 (1.05 to 1.1) | 1.09 (1.07 to 1.11) |  | 1.06 (1.04 to 1.08) | 1.06 (1.04 to 1.07) |  | 1.06 (1.04 to 1.07) | 1.07 (1.06 to 1.09) |
| **Ethnicity** | |  |  |  |  |  |  |  |  |
|  | White ^ref^ |  |  |  |  |  |  |  |  |
|  | Black | 0.94 (0.8 to 1.1) | 0.75 (0.64 to 0.86) |  | 0.89 (0.79 to 1.02) | 0.69 (0.62 to 0.78) |  | 0.86 (0.78 to 0.96) | 0.71 (0.64 to 0.78) |
|  | Asian | 0.58 (0.51 to 0.66) | 0.58 (0.53 to 0.64) |  | 0.64 (0.58 to 0.72) | 0.52 (0.48 to 0.57) |  | 0.66 (0.6 to 0.72) | 0.59 (0.55 to 0.63) |
|  | Mixed | 0.66 (0.51 to 0.86) | 0.54 (0.42 to 0.7) |  | 0.78 (0.62 to 0.98) | 0.58 (0.47 to 0.72) |  | 0.84 (0.69 to 1.01) | 0.58 (0.48 to 0.69) |
|  | Other | 0.57 (0.41 to 0.78) | 0.44 (0.35 to 0.56) |  | 0.56 (0.43 to 0.74) | 0.48 (0.4 to 0.58) |  | 0.53 (0.42 to 0.68) | 0.45 (0.38 to 0.54) |
|  | Unknown | 0.95 (0.78 to 1.16) | 0.65 (0.58 to 0.73) |  | 1.03 (0.88 to 1.22) | 0.66 (0.6 to 0.72) |  | 1.12 (0.99 to 1.27) | 0.8 (0.74 to 0.86) |
| **IMD Quintile** | |  |  |  |  |  |  |  |  |
|  | Q5 (least deprived)  ^ref^ |  |  |  |  |  |  |  |  |
|  | Q4 | 0.96 (0.84 to 1.09) | 1.17 (1.04 to 1.31) |  | 1.14 (1.02 to 1.27) | 1.17 (1.06 to 1.29) |  | 1.16 (1.07 to 1.26) | 1.19 (1.11 to 1.27) |
|  | Q3 | 1.14 (1 to 1.29) | 1.33 (1.19 to 1.48) |  | 1.28 (1.15 to 1.42) | 1.45 (1.32 to 1.58) |  | 1.32 (1.22 to 1.43) | 1.42 (1.33 to 1.52) |
|  | Q2 | 1.28 (1.14 to 1.45) | 1.77 (1.6 to 1.96) |  | 1.52 (1.37 to 1.68) | 1.72 (1.58 to 1.88) |  | 1.5 (1.39 to 1.62) | 1.65 (1.55 to 1.76) |
|  | Q1 (most deprived) | 1.7 (1.51 to 1.91) | 2.08 (1.87 to 2.3) |  | 1.8 (1.63 to 1.99) | 2.2 (2.02 to 2.39) |  | 1.7 (1.57 to 1.83) | 2.06 (1.93 to 2.19) |
|  | Unknown | 4.44 (3.48 to 5.66) | 6.92 (5.72 to 8.38) |  | 5.2 (4.17 to 6.48) | 6.46 (5.47 to 7.62) |  | 6.67 (5.64 to 7.89) | 6.75 (5.93 to 7.68) |
| **Region** | |  |  |  |  |  |  |  |  |
|  | London ^ref^ |  |  |  |  |  |  |  |  |
|  | East Midlands | 1.25 (1.07 to 1.45) | 1.78 (1.58 to 2) |  | 1.24 (1.1 to 1.41) | 1.52 (1.38 to 1.68) |  | 1.29 (1.18 to 1.42) | 1.34 (1.24 to 1.45) |
|  | East of England | 1.15 (0.99 to 1.32) | 1.41 (1.26 to 1.58) |  | 1.25 (1.11 to 1.4) | 1.2 (1.09 to 1.32) |  | 1.06 (0.97 to 1.17) | 1.18 (1.1 to 1.27) |
|  | North East | 1.68 (1.43 to 1.97) | 1.95 (1.72 to 2.22) |  | 1.29 (1.12 to 1.49) | 1.65 (1.48 to 1.84) |  | 1.22 (1.09 to 1.36) | 1.22 (1.12 to 1.34) |
|  | North West | 1.36 (1.19 to 1.54) | 1.46 (1.32 to 1.62) |  | 1.32 (1.19 to 1.47) | 1.35 (1.24 to 1.47) |  | 1.27 (1.17 to 1.38) | 1.27 (1.19 to 1.35) |
|  | South East | 1.3 (1.14 to 1.48) | 1.39 (1.24 to 1.55) |  | 1.17 (1.05 to 1.31) | 1.29 (1.18 to 1.41) |  | 1.1 (1.01 to 1.2) | 1.18 (1.1 to 1.27) |
|  | South West | 1.16 (1 to 1.35) | 1.51 (1.34 to 1.7) |  | 1.1 (0.97 to 1.25) | 1.35 (1.22 to 1.49) |  | 1.16 (1.06 to 1.28) | 1.19 (1.1 to 1.29) |
|  | West Midlands | 1.33 (1.16 to 1.52) | 1.72 (1.55 to 1.92) |  | 1.25 (1.12 to 1.4) | 1.44 (1.32 to 1.57) |  | 1.26 (1.16 to 1.38) | 1.33 (1.24 to 1.42) |
|  | Yorkshire Humber | 1.51 (1.32 to 1.73) | 1.65 (1.47 to 1.84) |  | 1.38 (1.24 to 1.55) | 1.6 (1.46 to 1.76) |  | 1.29 (1.18 to 1.41) | 1.35 (1.26 to 1.45) |
| **Health characteristics** | |  |  |  |  |  |  |  |  |
|  | Atrial fibrillation (QOF) | 1.32 (0.8 to 2.19) | 1.12 (0.83 to 1.53) |  | 1.56 (1.08 to 2.25) | 1.27 (1.04 to 1.56) |  | 1.25 (0.99 to 1.57) | 1.22 (1.07 to 1.39) |
|  | Asthma (QOF) | 0.87 (0.76 to 0.98) | 0.86 (0.76 to 0.97) |  | 1.02 (0.92 to 1.12) | 0.94 (0.86 to 1.03) |  | 0.86 (0.8 to 0.92) | 0.87 (0.81 to 0.94) |
|  | Cancer (QOF) | 10.14 (9.16 to 11.22) | 5.31 (4.7 to 6) |  | 7.89 (7.28 to 8.56) | 4.63 (4.2 to 5.1) |  | 5.91 (5.57 to 6.26) | 4.43 (4.14 to 4.73) |
|  | COPD (QOF) | 1.66 (1.21 to 2.29) | 1.58 (1.19 to 2.09) |  | 1.42 (1.18 to 1.71) | 1.6 (1.38 to 1.87) |  | 1.43 (1.29 to 1.59) | 1.48 (1.35 to 1.62) |
|  | Heart failure (QOF) | 1.41 (1 to 1.99) | 1.8 (1.4 to 2.33) |  | 1.84 (1.43 to 2.36) | 2.05 (1.74 to 2.43) |  | 1.83 (1.55 to 2.16) | 1.51 (1.34 to 1.71) |
|  | Palliative care (QOF) | 7.21 (6.18 to 8.42) | 8.44 (7.04 to 10.13) |  | 8.25 (7.3 to 9.32) | 8.21 (7.16 to 9.41) |  | 7.03 (6.45 to 7.65) | 6.09 (5.51 to 6.73) |
|  | Bipolar & schizophrenia (GPES) | 1.91 (1.56 to 2.33) | 2.61 (2.28 to 2.99) |  | 2.04 (1.75 to 2.37) | 2.28 (2.05 to 2.54) |  | 1.42 (1.25 to 1.6) | 2.01 (1.84 to 2.18) |
|  | Coronary heart disease (GPES) | 0.95 (0.59 to 1.53) | 0.67 (0.49 to 0.92) |  | 1.21 (0.95 to 1.55) | 0.87 (0.75 to 1.02) |  | 0.95 (0.82 to 1.11) | 0.87 (0.8 to 0.96) |
|  | Epilepsy (GPES) | 2.05 (1.75 to 2.4) | 2.39 (2.12 to 2.71) |  | 1.92 (1.68 to 2.19) | 1.93 (1.74 to 2.15) |  | 1.63 (1.47 to 1.81) | 1.92 (1.78 to 2.08) |
|  | Learning dis. & Downs (GPES) | 2.33 (1.91 to 2.86) | 1.88 (1.61 to 2.19) |  | 2.8 (2.37 to 3.31) | 1.69 (1.46 to 1.95) |  | 2.85 (2.51 to 3.25) | 1.71 (1.53 to 1.92) |
|  | Liver cirrhosis (GPES) | 6.72 (5.38 to 8.39) | 6.68 (5.52 to 8.1) |  | 7.09 (6.02 to 8.34) | 5.17 (4.52 to 5.92) |  | 5.71 (5.08 to 6.43) | 4.6 (4.19 to 5.06) |
|  | Stroke & TIA (GPES) | 1.59 (1.16 to 2.17) | 1.44 (1.11 to 1.86) |  | 1.17 (0.92 to 1.48) | 1.38 (1.16 to 1.65) |  | 1.3 (1.13 to 1.5) | 1.18 (1.05 to 1.32) |
|  | Chronic respiratory disease (HES) | 1.57 (1.41 to 1.76) | 1.83 (1.64 to 2.04) |  | 1.54 (1.4 to 1.7) | 1.75 (1.6 to 1.91) |  | 1.56 (1.45 to 1.68) | 1.62 (1.52 to 1.73) |
|  | Cardiovascular disease (HES) | 3.34 (3.04 to 3.68) | 4.19 (3.86 to 4.56) |  | 2.89 (2.68 to 3.12) | 3.52 (3.3 to 3.76) |  | 2.58 (2.44 to 2.72) | 2.73 (2.6 to 2.86) |
|  | Chronic kidney disease (QOF only) | 0.46 (0.21 to 1.04) | 1.21 (0.72 to 2.06) |  | 0.99 (0.66 to 1.48) | 1.64 (1.21 to 2.24) |  | 1.69 (1.39 to 2.05) | 1.49 (1.23 to 1.81) |
|  | Chronic kidney disease (HES) | 2.54 (2.09 to 3.09) | 1.88 (1.57 to 2.26) |  | 2.7 (2.32 to 3.16) | 2.15 (1.87 to 2.47) |  | 1.96 (1.75 to 2.19) | 2.21 (2.01 to 2.43) |
|  | Dementia (HES or QOF) | 4.7 (2.93 to 7.52) | 1.5 (0.67 to 3.36) |  | 3.27 (2.25 to 4.76) | 2.31 (1.54 to 3.47) |  | 3.04 (2.37 to 3.89) | 2.77 (2.21 to 3.47) |
|  | Diabetes (QOF only) | 0.79 (0.59 to 1.07) | 1.14 (0.93 to 1.4) |  | 0.97 (0.8 to 1.17) | 1.24 (1.08 to 1.43) |  | 1.04 (0.91 to 1.18) | 1.3 (1.19 to 1.42) |
|  | Diabetes (HES) | 1.53 (1.32 to 1.77) | 1.67 (1.46 to 1.92) |  | 1.45 (1.29 to 1.62) | 1.65 (1.5 to 1.82) |  | 1.28 (1.19 to 1.39) | 1.6 (1.5 to 1.71) |
|  | Clinically vulnerable (NIMS) | 2.52 (2.3 to 2.76) | 1.83 (1.65 to 2.03) |  | 2.19 (2.03 to 2.37) | 1.57 (1.45 to 1.7) |  | 2.29 (2.16 to 2.43) | 1.56 (1.47 to 1.65) |
|  | Care home resident (GPES) | 2.32 (1.28 to 4.2) | 2.2 (1.39 to 3.48) |  | 2.33 (1.51 to 3.6) | 3.62 (2.72 to 4.82) |  | 4.07 (3.19 to 5.2) | 1.42 (1.12 to 1.8) |
| **Smoking status** | |  |  |  |  |  |  |  |  |
|  | Never smoker (NHSD) ^ref^ |  |  |  |  |  |  |  |  |
|  | Current smoker (NHSD) | 1.9 (1.75 to 2.06) | 1.51 (1.42 to 1.61) |  | 1.91 (1.78 to 2.05) | 1.69 (1.6 to 1.78) |  | 1.85 (1.75 to 1.95) | 1.92 (1.85 to 2) |
|  | Ex-smoker (NHSD) | 0.94 (0.85 to 1.03) | 0.91 (0.84 to 0.99) |  | 0.99 (0.91 to 1.07) | 0.86 (0.81 to 0.92) |  | 1.03 (0.98 to 1.1) | 0.93 (0.89 to 0.98) |
|  | Non-smoker (NHSD) | 0.9 (0.75 to 1.08) | 0.82 (0.68 to 0.98) |  | 0.91 (0.77 to 1.07) | 0.83 (0.71 to 0.97) |  | 0.93 (0.82 to 1.06) | 0.99 (0.89 to 1.11) |
| **Wave & COVID-19 & Vaccination status** | |  |  |  |  |  |  |  |  |
|  | Wave 1 & 0-4 weeks | 48.73 (34.61 to 68.6) | 42.32 (31.12 to 57.55) |  | 45.83 (35.32 to 59.46) | 72.47 (59.55 to 88.2) |  | 51.69 (42.64 to 62.66) | 64.84 (55.58 to 75.65) |
|  | Wave 1 & 4+ weeks | 7.72 (4.76 to 12.52) | 4.91 (2.89 to 8.34) |  | 5.15 (3.34 to 7.96) | 6.77 (4.74 to 9.68) |  | 6.46 (4.78 to 8.73) | 7.46 (5.77 to 9.63) |
|  | Wave 2 & 0-4 weeks | 12.93 (10.39 to 16.08) | 13.48 (11.12 to 16.33) |  | 14.72 (12.32 to 17.58) | 13.73 (11.75 to 16.04) |  | 15.5 (13.53 to 17.76) | 17.51 (15.67 to 19.57) |
|  | Wave 2 & 4+ weeks | 2.5 (1.83 to 3.4) | 2.58 (1.99 to 3.36) |  | 2.88 (2.25 to 3.7) | 2.84 (2.3 to 3.5) |  | 3.11 (2.59 to 3.74) | 3.92 (3.39 to 4.52) |
|  | Wave 3 & 0-4 weeks | 11.18 (8.25 to 15.15) | 13.33 (10.5 to 16.93) |  | 13.94 (10.64 to 18.25) | 13.23 (10.56 to 16.57) |  | 11.99 (9.39 to 15.31) | 22.28 (18.81 to 26.39) |
|  | Wave 3 & 4+ weeks | 2.22 (1.7 to 2.91) | 2.67 (2.15 to 3.31) |  | 1.95 (1.47 to 2.58) | 3.07 (2.54 to 3.71) |  | 2.39 (1.92 to 2.97) | 3.57 (3.02 to 4.22) |
|  | 1st vaccine date | 0.89 (0.8 to 1) | 0.97 (0.89 to 1.06) |  | 0.91 (0.82 to 1) | 0.9 (0.83 to 0.96) |  | 0.75 (0.7 to 0.81) | 0.93 (0.88 to 0.99) |
|  | Wave 2 & 0-4 weeks & 1st vaccine | 0.22 (0.03 to 1.56) | 0.75 (0.18 to 3.05) |  | 0.45 (0.14 to 1.42) | 0.89 (0.33 to 2.4) |  | 0.78 (0.43 to 1.4) | 0.37 (0.14 to 0.98) |
|  | Wave 3 & 0-4 weeks & 1st vaccine | 0.13 (0.08 to 0.21) | 0.15 (0.11 to 0.22) |  | 0.12 (0.08 to 0.18) | 0.16 (0.12 to 0.22) |  | 0.23 (0.17 to 0.31) | 0.12 (0.1 to 0.16) |
|  | Wave 2 & 4+ weeks & 1st vaccine | 0.6 (0.25 to 1.43) | 0.25 (0.06 to 1.03) |  | 0.62 (0.32 to 1.18) | 0.69 (0.35 to 1.38) |  | 0.38 (0.22 to 0.68) | 0.34 (0.19 to 0.6) |
|  | Wave 3 & 4+ weeks & 1st vaccine | 0.49 (0.35 to 0.67) | 0.43 (0.33 to 0.56) |  | 0.49 (0.36 to 0.67) | 0.37 (0.29 to 0.46) |  | 0.41 (0.32 to 0.53) | 0.3 (0.24 to 0.36) |
|  |  |  |  |  |  |  |  |  |  |
|  |  | **50-54 years** | |  | **55-59 years** | |  | **60-64 years** | |
|  |  | **Female** | **Male** |  | **Female** | **Male** |  | **Female** | **Male** |
| **Age** | | 1.06 (1.04 to 1.07) | 1.06 (1.05 to 1.07) |  | 1.05 (1.04 to 1.06) | 1.06 (1.06 to 1.07) |  | 1.07 (1.06 to 1.08) | 1.06 (1.05 to 1.07) |
| **Ethnicity** | |  |  |  |  |  |  |  |  |
|  | White ^ref^ |  |  |  |  |  |  |  |  |
|  | Black | 0.91 (0.84 to 0.99) | 0.72 (0.67 to 0.77) |  | 0.88 (0.82 to 0.95) | 0.79 (0.74 to 0.84) |  | 0.84 (0.78 to 0.9) | 0.77 (0.72 to 0.81) |
|  | Asian | 0.67 (0.62 to 0.73) | 0.64 (0.6 to 0.68) |  | 0.68 (0.63 to 0.73) | 0.73 (0.69 to 0.77) |  | 0.75 (0.71 to 0.8) | 0.75 (0.72 to 0.79) |
|  | Mixed | 0.77 (0.65 to 0.92) | 0.67 (0.57 to 0.77) |  | 0.76 (0.65 to 0.88) | 0.76 (0.67 to 0.86) |  | 0.79 (0.68 to 0.91) | 0.8 (0.71 to 0.91) |
|  | Other | 0.66 (0.54 to 0.81) | 0.61 (0.53 to 0.7) |  | 0.51 (0.42 to 0.63) | 0.67 (0.59 to 0.76) |  | 0.57 (0.48 to 0.68) | 0.72 (0.64 to 0.81) |
|  | Unknown | 1.26 (1.15 to 1.38) | 0.89 (0.84 to 0.94) |  | 1.49 (1.38 to 1.6) | 1.04 (0.99 to 1.09) |  | 1.51 (1.42 to 1.61) | 1.08 (1.03 to 1.13) |
| **IMD Quintile** | |  |  |  |  |  |  |  |  |
|  | Q5 (least deprived)  ^ref^ |  |  |  |  |  |  |  |  |
|  | Q4 | 1.09 (1.03 to 1.16) | 1.13 (1.07 to 1.19) |  | 1.12 (1.06 to 1.18) | 1.13 (1.08 to 1.18) |  | 1.1 (1.06 to 1.15) | 1.1 (1.06 to 1.14) |
|  | Q3 | 1.22 (1.14 to 1.29) | 1.28 (1.21 to 1.34) |  | 1.21 (1.15 to 1.27) | 1.27 (1.22 to 1.32) |  | 1.21 (1.16 to 1.26) | 1.23 (1.19 to 1.27) |
|  | Q2 | 1.35 (1.27 to 1.43) | 1.5 (1.43 to 1.58) |  | 1.34 (1.28 to 1.41) | 1.45 (1.39 to 1.51) |  | 1.33 (1.28 to 1.39) | 1.4 (1.35 to 1.45) |
|  | Q1 (most deprived) | 1.53 (1.44 to 1.62) | 1.79 (1.7 to 1.88) |  | 1.47 (1.4 to 1.55) | 1.7 (1.64 to 1.77) |  | 1.47 (1.41 to 1.53) | 1.61 (1.56 to 1.67) |
|  | Unknown | 7.11 (6.21 to 8.14) | 4.98 (4.48 to 5.55) |  | 7.51 (6.73 to 8.39) | 4.92 (4.51 to 5.37) |  | 7.37 (6.7 to 8.1) | 4.44 (4.1 to 4.81) |
| **Region** | |  |  |  |  |  |  |  |  |
|  | London ^ref^ |  |  |  |  |  |  |  |  |
|  | East Midlands | 1.23 (1.14 to 1.32) | 1.17 (1.11 to 1.25) |  | 1.19 (1.12 to 1.27) | 1.08 (1.03 to 1.14) |  | 1.14 (1.08 to 1.2) | 1.12 (1.07 to 1.17) |
|  | East of England | 1.17 (1.09 to 1.26) | 1.13 (1.07 to 1.2) |  | 1.08 (1.02 to 1.15) | 1.05 (1.01 to 1.11) |  | 1.09 (1.03 to 1.15) | 1.05 (1 to 1.09) |
|  | North East | 1.15 (1.05 to 1.26) | 1.15 (1.07 to 1.23) |  | 1.14 (1.06 to 1.22) | 1.01 (0.96 to 1.07) |  | 1.1 (1.03 to 1.17) | 0.98 (0.93 to 1.03) |
|  | North West | 1.17 (1.09 to 1.25) | 1.16 (1.1 to 1.23) |  | 1.12 (1.06 to 1.19) | 1.04 (1 to 1.09) |  | 1.03 (0.98 to 1.08) | 1.03 (0.99 to 1.07) |
|  | South East | 1.2 (1.12 to 1.28) | 1.11 (1.05 to 1.17) |  | 1.14 (1.07 to 1.2) | 1.04 (1 to 1.09) |  | 1.1 (1.04 to 1.15) | 1.09 (1.05 to 1.13) |
|  | South West | 1.17 (1.09 to 1.26) | 1.13 (1.06 to 1.2) |  | 1.09 (1.02 to 1.16) | 1.01 (0.96 to 1.06) |  | 1.05 (0.99 to 1.11) | 1.05 (1.01 to 1.1) |
|  | West Midlands | 1.19 (1.11 to 1.28) | 1.17 (1.11 to 1.24) |  | 1.11 (1.04 to 1.18) | 1.09 (1.04 to 1.14) |  | 1.15 (1.09 to 1.21) | 1.13 (1.09 to 1.18) |
|  | Yorkshire Humber | 1.22 (1.13 to 1.31) | 1.2 (1.13 to 1.27) |  | 1.15 (1.08 to 1.22) | 1.07 (1.02 to 1.13) |  | 1.17 (1.11 to 1.24) | 1.13 (1.08 to 1.18) |
| **Health characteristics** | |  |  |  |  |  |  |  |  |
|  | Atrial fibrillation (QOF) | 1.16 (1 to 1.35) | 1.17 (1.08 to 1.27) |  | 1.32 (1.21 to 1.45) | 1.12 (1.06 to 1.19) |  | 1.14 (1.06 to 1.21) | 1.14 (1.1 to 1.19) |
|  | Asthma (QOF) | 0.82 (0.78 to 0.87) | 0.78 (0.74 to 0.83) |  | 0.8 (0.76 to 0.84) | 0.79 (0.76 to 0.83) |  | 0.74 (0.71 to 0.77) | 0.74 (0.71 to 0.77) |
|  | Cancer (QOF) | 4.58 (4.38 to 4.78) | 3.79 (3.61 to 3.97) |  | 3.51 (3.39 to 3.64) | 3.34 (3.22 to 3.45) |  | 2.84 (2.75 to 2.92) | 2.61 (2.54 to 2.68) |
|  | COPD (QOF) | 1.36 (1.26 to 1.46) | 1.35 (1.27 to 1.44) |  | 1.35 (1.28 to 1.42) | 1.38 (1.31 to 1.44) |  | 1.31 (1.25 to 1.37) | 1.34 (1.29 to 1.39) |
|  | Heart failure (QOF) | 1.61 (1.44 to 1.81) | 1.89 (1.74 to 2.04) |  | 1.6 (1.47 to 1.75) | 1.62 (1.53 to 1.72) |  | 1.68 (1.58 to 1.8) | 1.62 (1.55 to 1.7) |
|  | Palliative care (QOF) | 7.69 (7.21 to 8.19) | 5.66 (5.28 to 6.08) |  | 6.95 (6.59 to 7.32) | 5.14 (4.87 to 5.41) |  | 5.79 (5.54 to 6.05) | 4.66 (4.47 to 4.86) |
|  | Bipolar & schizophrenia (GPES) | 1.54 (1.4 to 1.69) | 1.77 (1.64 to 1.9) |  | 1.52 (1.41 to 1.65) | 1.66 (1.56 to 1.76) |  | 1.43 (1.33 to 1.53) | 1.44 (1.35 to 1.53) |
|  | Coronary heart disease (GPES) | 0.98 (0.89 to 1.08) | 0.89 (0.84 to 0.95) |  | 0.96 (0.9 to 1.02) | 0.92 (0.88 to 0.95) |  | 0.95 (0.91 to 1) | 0.95 (0.92 to 0.98) |
|  | Epilepsy (GPES) | 1.47 (1.35 to 1.6) | 1.68 (1.57 to 1.79) |  | 1.35 (1.26 to 1.45) | 1.49 (1.41 to 1.58) |  | 1.33 (1.25 to 1.42) | 1.41 (1.34 to 1.49) |
|  | Learning dis. & Downs (GPES) | 2.51 (2.25 to 2.79) | 2.1 (1.93 to 2.29) |  | 2.82 (2.58 to 3.08) | 2.03 (1.88 to 2.19) |  | 2.06 (1.89 to 2.26) | 2 (1.86 to 2.15) |
|  | Liver cirrhosis (GPES) | 4.16 (3.77 to 4.59) | 4.21 (3.92 to 4.52) |  | 3.43 (3.16 to 3.72) | 3 (2.82 to 3.19) |  | 2.57 (2.39 to 2.77) | 2.71 (2.57 to 2.86) |
|  | Stroke & TIA (GPES) | 1.23 (1.12 to 1.35) | 1.28 (1.19 to 1.37) |  | 1.29 (1.2 to 1.38) | 1.25 (1.19 to 1.32) |  | 1.13 (1.07 to 1.19) | 1.17 (1.13 to 1.22) |
|  | Chronic respiratory disease (HES) | 1.53 (1.44 to 1.62) | 1.59 (1.52 to 1.68) |  | 1.4 (1.34 to 1.47) | 1.49 (1.43 to 1.55) |  | 1.48 (1.42 to 1.54) | 1.43 (1.38 to 1.48) |
|  | Cardiovascular disease (HES) | 2.22 (2.13 to 2.32) | 2.24 (2.16 to 2.32) |  | 2.05 (1.98 to 2.12) | 1.91 (1.86 to 1.97) |  | 1.9 (1.85 to 1.96) | 1.74 (1.7 to 1.78) |
|  | Chronic kidney disease (QOF only) | 1.29 (1.14 to 1.47) | 1.26 (1.11 to 1.43) |  | 1.23 (1.13 to 1.34) | 1.23 (1.13 to 1.34) |  | 1.14 (1.07 to 1.21) | 1.19 (1.12 to 1.26) |
|  | Chronic kidney disease (HES) | 2.33 (2.15 to 2.52) | 1.98 (1.85 to 2.12) |  | 1.98 (1.86 to 2.11) | 2.1 (1.99 to 2.21) |  | 1.93 (1.84 to 2.03) | 1.86 (1.79 to 1.94) |
|  | Dementia (HES or QOF) | 3.25 (2.8 to 3.77) | 2.36 (2.05 to 2.72) |  | 2.74 (2.47 to 3.04) | 2.14 (1.95 to 2.35) |  | 2.75 (2.55 to 2.95) | 2.16 (2.03 to 2.3) |
|  | Diabetes (QOF only) | 1.14 (1.04 to 1.25) | 1.23 (1.15 to 1.3) |  | 1.07 (0.99 to 1.15) | 1.25 (1.19 to 1.31) |  | 1.25 (1.18 to 1.32) | 1.22 (1.17 to 1.27) |
|  | Diabetes (HES) | 1.34 (1.26 to 1.42) | 1.48 (1.42 to 1.56) |  | 1.37 (1.31 to 1.43) | 1.43 (1.38 to 1.48) |  | 1.39 (1.34 to 1.44) | 1.4 (1.36 to 1.44) |
|  | Clinically vulnerable (NIMS) | 2.24 (2.14 to 2.34) | 1.63 (1.57 to 1.7) |  | 2.16 (2.08 to 2.24) | 1.61 (1.56 to 1.66) |  | 1.91 (1.85 to 1.97) | 1.58 (1.54 to 1.62) |
|  | Care home resident (GPES) | 3.79 (3.19 to 4.51) | 1.56 (1.33 to 1.83) |  | 1.97 (1.72 to 2.26) | 2.36 (2.13 to 2.62) |  | 2.24 (2.04 to 2.46) | 2.25 (2.08 to 2.43) |
| **Smoking status** | |  |  |  |  |  |  |  |  |
|  | Never smoker (NHSD) ^ref^ |  |  |  |  |  |  |  |  |
|  | Current smoker (NHSD) | 1.79 (1.71 to 1.87) | 1.95 (1.89 to 2.02) |  | 1.84 (1.78 to 1.91) | 1.92 (1.87 to 1.98) |  | 1.85 (1.79 to 1.91) | 1.9 (1.85 to 1.95) |
|  | Ex-smoker (NHSD) | 1.04 (0.99 to 1.08) | 0.99 (0.96 to 1.03) |  | 1.14 (1.1 to 1.19) | 1.05 (1.02 to 1.08) |  | 1.15 (1.11 to 1.18) | 1.11 (1.08 to 1.14) |
|  | Non-smoker (NHSD) | 0.91 (0.82 to 1.01) | 0.92 (0.84 to 1.01) |  | 0.98 (0.9 to 1.07) | 0.92 (0.85 to 1) |  | 1.02 (0.95 to 1.1) | 0.97 (0.91 to 1.04) |
| **Wave & COVID-19 & Vaccination status** | |  |  |  |  |  |  |  |  |
|  | Wave 1 & 0-4 weeks | 58.22 (50.48 to 67.15) | 78.82 (70.47 to 88.15) |  | 58.2 (51.71 to 65.51) | 90.07 (83.05 to 97.69) |  | 64.22 (58.38 to 70.65) | 80.7 (75.22 to 86.58) |
|  | Wave 1 & 4+ weeks | 5.53 (4.27 to 7.15) | 6.75 (5.5 to 8.29) |  | 4.51 (3.59 to 5.67) | 6.99 (5.96 to 8.2) |  | 5.17 (4.32 to 6.2) | 6.94 (6.08 to 7.93) |
|  | Wave 2 & 0-4 weeks | 19.22 (17.39 to 21.24) | 20.45 (18.83 to 22.21) |  | 22.48 (20.66 to 24.46) | 25.23 (23.63 to 26.95) |  | 28.46 (26.54 to 30.53) | 34.02 (32.23 to 35.91) |
|  | Wave 2 & 4+ weeks | 3.42 (2.96 to 3.96) | 3.87 (3.45 to 4.35) |  | 4.44 (3.94 to 5) | 5.6 (5.13 to 6.11) |  | 5.38 (4.86 to 5.97) | 6.37 (5.89 to 6.9) |
|  | Wave 3 & 0-4 weeks | 17.27 (14.21 to 20.99) | 26.98 (23.33 to 31.21) |  | 20.64 (17.42 to 24.45) | 29.64 (25.82 to 34.02) |  | 31.02 (26.65 to 36.1) | 42.21 (37.21 to 47.88) |
|  | Wave 3 & 4+ weeks | 2.34 (1.9 to 2.88) | 3.85 (3.3 to 4.49) |  | 2.9 (2.43 to 3.48) | 4.36 (3.78 to 5.03) |  | 3.81 (3.22 to 4.51) | 5.8 (5.08 to 6.63) |
|  | 1st vaccine date | 0.69 (0.65 to 0.74) | 0.86 (0.82 to 0.91) |  | 0.75 (0.71 to 0.79) | 0.88 (0.84 to 0.92) |  | 0.76 (0.72 to 0.8) | 0.89 (0.85 to 0.93) |
|  | Wave 2 & 0-4 weeks & 1st vaccine | 0.73 (0.46 to 1.15) | 1 (0.65 to 1.54) |  | 0.97 (0.72 to 1.32) | 0.81 (0.59 to 1.11) |  | 0.67 (0.51 to 0.88) | 0.8 (0.63 to 1) |
|  | Wave 3 & 0-4 weeks & 1st vaccine | 0.15 (0.11 to 0.19) | 0.11 (0.09 to 0.14) |  | 0.17 (0.14 to 0.2) | 0.14 (0.12 to 0.17) |  | 0.16 (0.13 to 0.19) | 0.13 (0.11 to 0.15) |
|  | Wave 2 & 4+ weeks & 1st vaccine | 0.25 (0.15 to 0.42) | 0.48 (0.34 to 0.69) |  | 0.24 (0.16 to 0.35) | 0.24 (0.17 to 0.33) |  | 0.25 (0.18 to 0.34) | 0.29 (0.23 to 0.36) |
|  | Wave 3 & 4+ weeks & 1st vaccine | 0.47 (0.37 to 0.59) | 0.29 (0.24 to 0.34) |  | 0.38 (0.31 to 0.47) | 0.31 (0.27 to 0.36) |  | 0.36 (0.3 to 0.44) | 0.26 (0.23 to 0.3) |
|  |  |  |  |  |  |  |  |  |  |
|  |  | **65-69 years** | |  | **70-74 years** | |  | **75-79 years** | |
|  |  | **Female** | **Male** |  | **Female** | **Male** |  | **Female** | **Male** |
| **Age** | | 1.07 (1.07 to 1.08) | 1.07 (1.06 to 1.07) |  | 1.08 (1.08 to 1.09) | 1.08 (1.07 to 1.08) |  | 1.08 (1.08 to 1.09) | 1.08 (1.08 to 1.09) |
| **Ethnicity** | |  |  |  |  |  |  |  |  |
|  | White ^ref^ |  |  |  |  |  |  |  |  |
|  | Black | 0.8 (0.74 to 0.86) | 0.74 (0.69 to 0.79) |  | 0.78 (0.73 to 0.84) | 0.78 (0.72 to 0.83) |  | 0.74 (0.7 to 0.78) | 0.8 (0.76 to 0.85) |
|  | Asian | 0.77 (0.73 to 0.81) | 0.74 (0.71 to 0.77) |  | 0.83 (0.79 to 0.86) | 0.75 (0.72 to 0.78) |  | 0.87 (0.84 to 0.91) | 0.82 (0.79 to 0.85) |
|  | Mixed | 0.73 (0.62 to 0.85) | 0.76 (0.67 to 0.86) |  | 0.75 (0.65 to 0.87) | 0.84 (0.74 to 0.95) |  | 0.82 (0.73 to 0.93) | 0.81 (0.72 to 0.9) |
|  | Other | 0.67 (0.57 to 0.78) | 0.62 (0.55 to 0.7) |  | 0.67 (0.58 to 0.77) | 0.75 (0.67 to 0.84) |  | 0.71 (0.63 to 0.81) | 0.71 (0.63 to 0.8) |
|  | Unknown | 1.73 (1.64 to 1.83) | 1.38 (1.32 to 1.44) |  | 1.89 (1.81 to 1.97) | 1.56 (1.5 to 1.62) |  | 1.84 (1.77 to 1.92) | 1.61 (1.55 to 1.67) |
| **IMD Quintile** | |  |  |  |  |  |  |  |  |
|  | Q5 (least deprived)  ^ref^ |  |  |  |  |  |  |  |  |
|  | Q4 | 1.07 (1.03 to 1.1) | 1.11 (1.08 to 1.14) |  | 1.09 (1.06 to 1.11) | 1.12 (1.09 to 1.14) |  | 1.06 (1.04 to 1.09) | 1.09 (1.07 to 1.12) |
|  | Q3 | 1.14 (1.1 to 1.18) | 1.23 (1.2 to 1.27) |  | 1.17 (1.14 to 1.2) | 1.21 (1.19 to 1.24) |  | 1.16 (1.13 to 1.18) | 1.18 (1.15 to 1.2) |
|  | Q2 | 1.27 (1.23 to 1.32) | 1.37 (1.33 to 1.41) |  | 1.27 (1.24 to 1.31) | 1.34 (1.31 to 1.37) |  | 1.22 (1.19 to 1.25) | 1.28 (1.25 to 1.31) |
|  | Q1 (most deprived) | 1.4 (1.35 to 1.45) | 1.59 (1.55 to 1.64) |  | 1.42 (1.38 to 1.46) | 1.49 (1.46 to 1.53) |  | 1.35 (1.31 to 1.38) | 1.4 (1.37 to 1.43) |
|  | Unknown | 7.16 (6.59 to 7.77) | 5.33 (4.98 to 5.71) |  | 8.74 (8.19 to 9.33) | 6.6 (6.24 to 6.99) |  | 8.41 (7.93 to 8.91) | 7.1 (6.73 to 7.48) |
| **Region** | |  |  |  |  |  |  |  |  |
|  | London ^ref^ |  |  |  |  |  |  |  |  |
|  | East Midlands | 1.15 (1.09 to 1.2) | 1.1 (1.06 to 1.14) |  | 1.17 (1.12 to 1.21) | 1.1 (1.06 to 1.13) |  | 1.09 (1.06 to 1.13) | 1.11 (1.08 to 1.14) |
|  | East of England | 1.11 (1.06 to 1.16) | 1.08 (1.04 to 1.12) |  | 1.16 (1.12 to 1.2) | 1.07 (1.04 to 1.1) |  | 1.13 (1.09 to 1.16) | 1.07 (1.04 to 1.1) |
|  | North East | 1.08 (1.02 to 1.14) | 1 (0.96 to 1.05) |  | 1.13 (1.08 to 1.17) | 1.01 (0.98 to 1.05) |  | 1.05 (1.01 to 1.09) | 1.05 (1.02 to 1.09) |
|  | North West | 1.03 (0.99 to 1.08) | 1.02 (0.99 to 1.06) |  | 1.1 (1.06 to 1.14) | 1 (0.98 to 1.03) |  | 1.04 (1.01 to 1.07) | 1.03 (1 to 1.06) |
|  | South East | 1.13 (1.08 to 1.18) | 1.06 (1.02 to 1.1) |  | 1.11 (1.08 to 1.15) | 1.06 (1.03 to 1.09) |  | 1.05 (1.02 to 1.08) | 1.06 (1.04 to 1.09) |
|  | South West | 1.04 (1 to 1.09) | 1 (0.96 to 1.04) |  | 1.08 (1.04 to 1.12) | 1.03 (1 to 1.06) |  | 1.06 (1.02 to 1.09) | 1.02 (0.99 to 1.05) |
|  | West Midlands | 1.1 (1.05 to 1.15) | 1.11 (1.07 to 1.15) |  | 1.16 (1.12 to 1.21) | 1.08 (1.05 to 1.11) |  | 1.09 (1.06 to 1.13) | 1.08 (1.05 to 1.11) |
|  | Yorkshire Humber | 1.17 (1.12 to 1.22) | 1.1 (1.06 to 1.14) |  | 1.2 (1.16 to 1.25) | 1.11 (1.08 to 1.15) |  | 1.16 (1.13 to 1.2) | 1.12 (1.09 to 1.15) |
| **Health characteristics** | |  |  |  |  |  |  |  |  |
|  | Atrial fibrillation (QOF) | 1.2 (1.15 to 1.25) | 1.11 (1.08 to 1.15) |  | 1.14 (1.11 to 1.17) | 1.11 (1.08 to 1.13) |  | 1.18 (1.15 to 1.2) | 1.1 (1.08 to 1.12) |
|  | Asthma (QOF) | 0.75 (0.73 to 0.78) | 0.72 (0.7 to 0.74) |  | 0.75 (0.73 to 0.77) | 0.69 (0.67 to 0.71) |  | 0.74 (0.72 to 0.76) | 0.73 (0.71 to 0.74) |
|  | Cancer (QOF) | 2.34 (2.28 to 2.4) | 2.11 (2.06 to 2.15) |  | 1.98 (1.94 to 2.02) | 1.76 (1.73 to 1.79) |  | 1.69 (1.66 to 1.72) | 1.47 (1.45 to 1.49) |
|  | COPD (QOF) | 1.34 (1.29 to 1.38) | 1.24 (1.2 to 1.28) |  | 1.35 (1.32 to 1.39) | 1.3 (1.27 to 1.33) |  | 1.43 (1.39 to 1.46) | 1.33 (1.3 to 1.36) |
|  | Heart failure (QOF) | 1.54 (1.47 to 1.62) | 1.62 (1.56 to 1.67) |  | 1.58 (1.53 to 1.64) | 1.56 (1.52 to 1.6) |  | 1.54 (1.5 to 1.59) | 1.5 (1.47 to 1.54) |
|  | Palliative care (QOF) | 4.65 (4.47 to 4.83) | 3.86 (3.73 to 3.99) |  | 3.81 (3.7 to 3.92) | 3.56 (3.47 to 3.66) |  | 2.93 (2.86 to 3.01) | 2.94 (2.87 to 3.01) |
|  | Bipolar & schizophrenia (GPES) | 1.39 (1.31 to 1.48) | 1.38 (1.3 to 1.45) |  | 1.2 (1.14 to 1.27) | 1.24 (1.18 to 1.31) |  | 1.13 (1.07 to 1.19) | 1.21 (1.14 to 1.28) |
|  | Coronary heart disease (GPES) | 0.96 (0.93 to 1) | 0.93 (0.91 to 0.96) |  | 0.94 (0.92 to 0.97) | 0.94 (0.92 to 0.96) |  | 0.96 (0.94 to 0.98) | 0.95 (0.93 to 0.96) |
|  | Epilepsy (GPES) | 1.33 (1.26 to 1.41) | 1.21 (1.15 to 1.27) |  | 1.2 (1.14 to 1.25) | 1.23 (1.18 to 1.28) |  | 1.13 (1.08 to 1.18) | 1.12 (1.08 to 1.17) |
|  | Learning dis. & Downs (GPES) | 2.05 (1.88 to 2.24) | 1.84 (1.71 to 1.98) |  | 1.71 (1.57 to 1.87) | 1.49 (1.38 to 1.6) |  | 1.5 (1.36 to 1.65) | 1.46 (1.34 to 1.59) |
|  | Liver cirrhosis (GPES) | 2.16 (2.02 to 2.31) | 2.33 (2.21 to 2.46) |  | 2.03 (1.92 to 2.16) | 2.14 (2.04 to 2.25) |  | 1.87 (1.76 to 1.98) | 1.97 (1.86 to 2.08) |
|  | Stroke & TIA (GPES) | 1.2 (1.16 to 1.25) | 1.2 (1.17 to 1.24) |  | 1.14 (1.11 to 1.18) | 1.16 (1.13 to 1.19) |  | 1.13 (1.11 to 1.16) | 1.1 (1.08 to 1.12) |
|  | Chronic respiratory disease (HES) | 1.52 (1.47 to 1.57) | 1.48 (1.43 to 1.52) |  | 1.47 (1.43 to 1.51) | 1.49 (1.46 to 1.52) |  | 1.47 (1.44 to 1.51) | 1.48 (1.45 to 1.51) |
|  | Cardiovascular disease (HES) | 1.79 (1.75 to 1.84) | 1.61 (1.58 to 1.64) |  | 1.68 (1.64 to 1.71) | 1.56 (1.53 to 1.58) |  | 1.57 (1.54 to 1.59) | 1.5 (1.48 to 1.52) |
|  | Chronic kidney disease (QOF only) | 1.07 (1.02 to 1.12) | 1.08 (1.04 to 1.12) |  | 1.02 (0.99 to 1.05) | 1.06 (1.03 to 1.09) |  | 1 (0.98 to 1.02) | 1.04 (1.02 to 1.06) |
|  | Chronic kidney disease (HES) | 1.63 (1.57 to 1.69) | 1.69 (1.64 to 1.75) |  | 1.55 (1.5 to 1.59) | 1.58 (1.54 to 1.62) |  | 1.46 (1.43 to 1.49) | 1.54 (1.51 to 1.57) |
|  | Dementia (HES or QOF) | 2.39 (2.27 to 2.51) | 2.27 (2.18 to 2.37) |  | 2.86 (2.78 to 2.95) | 2.63 (2.56 to 2.71) |  | 2.94 (2.88 to 3) | 2.77 (2.71 to 2.82) |
|  | Diabetes (QOF only) | 1.2 (1.14 to 1.26) | 1.22 (1.18 to 1.26) |  | 1.24 (1.2 to 1.29) | 1.21 (1.18 to 1.24) |  | 1.2 (1.16 to 1.24) | 1.18 (1.15 to 1.21) |
|  | Diabetes (HES) | 1.4 (1.36 to 1.44) | 1.41 (1.38 to 1.44) |  | 1.37 (1.34 to 1.4) | 1.36 (1.33 to 1.38) |  | 1.31 (1.28 to 1.33) | 1.31 (1.29 to 1.34) |
|  | Clinically vulnerable (NIMS) | 1.66 (1.62 to 1.71) | 1.43 (1.4 to 1.46) |  | 1.38 (1.35 to 1.41) | 1.19 (1.17 to 1.21) |  | 0.98 (0.97 to 1) | 0.86 (0.85 to 0.88) |
|  | Care home resident (GPES) | 2.02 (1.88 to 2.17) | 1.93 (1.82 to 2.05) |  | 1.78 (1.7 to 1.86) | 1.76 (1.69 to 1.83) |  | 1.67 (1.62 to 1.72) | 1.61 (1.56 to 1.66) |
| **Smoking status** | |  |  |  |  |  |  |  |  |
|  | Never smoker (NHSD) ^ref^ |  |  |  |  |  |  |  |  |
|  | Current smoker (NHSD) | 1.89 (1.84 to 1.95) | 1.92 (1.88 to 1.97) |  | 1.89 (1.84 to 1.93) | 1.78 (1.75 to 1.82) |  | 1.72 (1.68 to 1.76) | 1.57 (1.54 to 1.61) |
|  | Ex-smoker (NHSD) | 1.13 (1.1 to 1.16) | 1.11 (1.09 to 1.13) |  | 1.13 (1.11 to 1.15) | 1.06 (1.04 to 1.07) |  | 1.07 (1.05 to 1.09) | 1.01 (0.99 to 1.02) |
|  | Non-smoker (NHSD) | 0.97 (0.9 to 1.04) | 1.01 (0.95 to 1.07) |  | 1 (0.95 to 1.06) | 1 (0.95 to 1.05) |  | 1.02 (0.98 to 1.07) | 0.99 (0.95 to 1.03) |
| **Wave & COVID-19 & Vaccination status** | |  |  |  |  |  |  |  |  |
|  | Wave 1 & 0-4 weeks | 78.48 (72.42 to 85.05) | 80.26 (75.58 to 85.22) |  | 58.28 (54.69 to 62.11) | 70.35 (67.07 to 73.8) |  | 45.69 (43.39 to 48.11) | 55.39 (53.14 to 57.73) |
|  | Wave 1 & 4+ weeks | 7.86 (6.84 to 9.04) | 7.12 (6.35 to 7.98) |  | 5.65 (5.05 to 6.31) | 5.53 (5.02 to 6.08) |  | 4.32 (3.93 to 4.76) | 5.21 (4.8 to 5.66) |
|  | Wave 2 & 0-4 weeks | 39.54 (37.3 to 41.92) | 43.53 (41.56 to 45.6) |  | 41.98 (40.09 to 43.97) | 51.41 (49.5 to 53.38) |  | 38.44 (36.93 to 40.01) | 51.08 (49.39 to 52.83) |
|  | Wave 2 & 4+ weeks | 6.77 (6.17 to 7.42) | 7.78 (7.24 to 8.36) |  | 6.79 (6.29 to 7.33) | 8.55 (8.03 to 9.11) |  | 6.54 (6.11 to 6.99) | 8.02 (7.55 to 8.52) |
|  | Wave 3 & 0-4 weeks | 34.52 (29.88 to 39.88) | 43.14 (38.16 to 48.77) |  | 34.63 (30.23 to 39.66) | 48.51 (43.19 to 54.48) |  | 33.2 (29.37 to 37.54) | 44.2 (39.07 to 50) |
|  | Wave 3 & 4+ weeks | 5.05 (4.34 to 5.88) | 5.66 (4.94 to 6.48) |  | 4.59 (3.96 to 5.32) | 6.14 (5.37 to 7.02) |  | 4.43 (3.85 to 5.09) | 6.32 (5.5 to 7.25) |
|  | 1st vaccine date | 0.77 (0.74 to 0.81) | 0.86 (0.83 to 0.9) |  | 0.77 (0.73 to 0.8) | 0.92 (0.89 to 0.96) |  | 0.79 (0.76 to 0.81) | 0.94 (0.9 to 0.97) |
|  | Wave 2 & 0-4 weeks & 1st vaccine | 0.75 (0.6 to 0.93) | 0.66 (0.56 to 0.79) |  | 0.69 (0.59 to 0.8) | 0.68 (0.61 to 0.77) |  | 0.83 (0.75 to 0.91) | 0.73 (0.67 to 0.8) |
|  | Wave 3 & 0-4 weeks & 1st vaccine | 0.19 (0.16 to 0.22) | 0.15 (0.13 to 0.17) |  | 0.2 (0.17 to 0.23) | 0.16 (0.14 to 0.18) |  | 0.24 (0.21 to 0.28) | 0.21 (0.19 to 0.24) |
|  | Wave 2 & 4+ weeks & 1st vaccine | 0.3 (0.24 to 0.38) | 0.23 (0.19 to 0.29) |  | 0.32 (0.27 to 0.38) | 0.29 (0.25 to 0.34) |  | 0.41 (0.36 to 0.46) | 0.35 (0.31 to 0.4) |
|  | Wave 3 & 4+ weeks & 1st vaccine | 0.36 (0.31 to 0.43) | 0.31 (0.26 to 0.35) |  | 0.44 (0.37 to 0.51) | 0.35 (0.3 to 0.4) |  | 0.54 (0.47 to 0.62) | 0.38 (0.33 to 0.43) |
|  |  |  |  |  |  |  |  |  |  |
|  |  | **80-84 years** | |  | **85-89 years** | |  | **90+ years** | |
|  |  | **Female** | **Male** |  | **Female** | **Male** |  | **Female** | **Male** |
| **Age** | | 1.1 (1.09 to 1.1) | 1.1 (1.09 to 1.1) |  | 1.11 (1.11 to 1.11) | 1.11 (1.11 to 1.12) |  | 1.07 (1.07 to 1.08) | 1.08 (1.08 to 1.09) |
| **Ethnicity** | |  |  |  |  |  |  |  |  |
|  | White ^ref^ |  |  |  |  |  |  |  |  |
|  | Black | 0.75 (0.71 to 0.79) | 0.9 (0.86 to 0.94) |  | 0.78 (0.78 to 0.78) | 0.89 (0.84 to 0.93) |  | 0.87 (0.83 to 0.92) | 0.9 (0.84 to 0.96) |
|  | Asian | 0.9 (0.87 to 0.93) | 0.86 (0.83 to 0.89) |  | 0.88 (0.88 to 0.88) | 0.94 (0.91 to 0.97) |  | 0.84 (0.81 to 0.88) | 0.82 (0.78 to 0.86) |
|  | Mixed | 0.84 (0.76 to 0.94) | 0.95 (0.85 to 1.05) |  | 0.91 (0.91 to 0.91) | 0.92 (0.82 to 1.04) |  | 0.92 (0.83 to 1.01) | 0.98 (0.86 to 1.12) |
|  | Other | 0.7 (0.62 to 0.79) | 0.8 (0.71 to 0.91) |  | 0.7 (0.7 to 0.7) | 0.83 (0.72 to 0.94) |  | 0.85 (0.76 to 0.94) | 0.81 (0.69 to 0.94) |
|  | Unknown | 1.72 (1.66 to 1.78) | 1.67 (1.61 to 1.74) |  | 1.6 (1.6 to 1.6) | 1.48 (1.41 to 1.54) |  | 1.2 (1.16 to 1.24) | 0.62 (0.59 to 0.66) |
| **IMD Quintile** | |  |  |  |  |  |  |  |  |
|  | Q5 (least deprived)  ^ref^ |  |  |  |  |  |  |  |  |
|  | Q4 | 1.07 (1.05 to 1.09) | 1.08 (1.06 to 1.1) |  | 1.07 (1.07 to 1.07) | 1.08 (1.06 to 1.1) |  | 1.07 (1.06 to 1.09) | 1.09 (1.07 to 1.11) |
|  | Q3 | 1.13 (1.11 to 1.15) | 1.15 (1.12 to 1.17) |  | 1.11 (1.11 to 1.11) | 1.14 (1.12 to 1.17) |  | 1.11 (1.09 to 1.13) | 1.16 (1.13 to 1.18) |
|  | Q2 | 1.2 (1.18 to 1.23) | 1.25 (1.23 to 1.27) |  | 1.17 (1.17 to 1.17) | 1.23 (1.2 to 1.25) |  | 1.15 (1.13 to 1.16) | 1.21 (1.18 to 1.23) |
|  | Q1 (most deprived) | 1.29 (1.26 to 1.31) | 1.37 (1.34 to 1.4) |  | 1.24 (1.24 to 1.24) | 1.33 (1.3 to 1.36) |  | 1.18 (1.16 to 1.2) | 1.27 (1.24 to 1.3) |
|  | Unknown | 7.02 (6.68 to 7.38) | 5.77 (5.48 to 6.06) |  | 6.07 (6.07 to 6.07) | 4.77 (4.52 to 5.03) |  | 3.89 (3.72 to 4.06) | 3.31 (3.11 to 3.52) |
| **Region** | |  |  |  |  |  |  |  |  |
|  | London ^ref^ |  |  |  |  |  |  |  |  |
|  | East Midlands | 1.1 (1.07 to 1.13) | 1.1 (1.07 to 1.13) |  | 1.11 (1.11 to 1.11) | 1.04 (1.01 to 1.07) |  | 1.07 (1.05 to 1.1) | 1.06 (1.02 to 1.09) |
|  | East of England | 1.12 (1.09 to 1.15) | 1.09 (1.06 to 1.12) |  | 1.14 (1.14 to 1.14) | 1.06 (1.04 to 1.09) |  | 1.14 (1.11 to 1.16) | 1.09 (1.05 to 1.12) |
|  | North East | 1.08 (1.04 to 1.11) | 1.05 (1.02 to 1.08) |  | 1.05 (1.05 to 1.05) | 1.01 (0.98 to 1.04) |  | 1.1 (1.07 to 1.13) | 1.01 (0.97 to 1.05) |
|  | North West | 1.03 (1 to 1.05) | 1.03 (1.01 to 1.06) |  | 1.04 (1.04 to 1.04) | 1 (0.97 to 1.02) |  | 1.03 (1.01 to 1.06) | 1 (0.97 to 1.03) |
|  | South East | 1.04 (1.02 to 1.07) | 1.07 (1.05 to 1.1) |  | 1.04 (1.04 to 1.04) | 1.05 (1.03 to 1.08) |  | 1.07 (1.04 to 1.09) | 1.08 (1.05 to 1.11) |
|  | South West | 1.09 (1.06 to 1.12) | 1.06 (1.03 to 1.09) |  | 1.08 (1.08 to 1.08) | 1.04 (1.01 to 1.07) |  | 1.11 (1.08 to 1.13) | 1.06 (1.03 to 1.1) |
|  | West Midlands | 1.06 (1.03 to 1.09) | 1.06 (1.03 to 1.09) |  | 1.04 (1.04 to 1.04) | 1.03 (1 to 1.06) |  | 1.04 (1.02 to 1.07) | 1.03 (1 to 1.07) |
|  | Yorkshire Humber | 1.15 (1.12 to 1.18) | 1.12 (1.09 to 1.15) |  | 1.15 (1.15 to 1.15) | 1.08 (1.05 to 1.11) |  | 1.15 (1.13 to 1.18) | 1.08 (1.05 to 1.12) |
| **Health characteristics** | |  |  |  |  |  |  |  |  |
|  | Atrial fibrillation (QOF) | 1.17 (1.15 to 1.19) | 1.07 (1.06 to 1.09) |  | 1.17 (1.17 to 1.17) | 1.09 (1.07 to 1.1) |  | 1.15 (1.14 to 1.16) | 1.1 (1.08 to 1.12) |
|  | Asthma (QOF) | 0.76 (0.74 to 0.77) | 0.76 (0.75 to 0.78) |  | 0.8 (0.8 to 0.8) | 0.77 (0.75 to 0.79) |  | 0.82 (0.8 to 0.84) | 0.82 (0.8 to 0.85) |
|  | Cancer (QOF) | 1.46 (1.44 to 1.48) | 1.33 (1.31 to 1.34) |  | 1.27 (1.27 to 1.27) | 1.21 (1.19 to 1.23) |  | 1.11 (1.09 to 1.13) | 1.11 (1.09 to 1.13) |
|  | COPD (QOF) | 1.42 (1.38 to 1.45) | 1.33 (1.3 to 1.36) |  | 1.36 (1.36 to 1.36) | 1.24 (1.21 to 1.26) |  | 1.28 (1.25 to 1.31) | 1.19 (1.16 to 1.23) |
|  | Heart failure (QOF) | 1.49 (1.46 to 1.53) | 1.53 (1.5 to 1.56) |  | 1.41 (1.41 to 1.41) | 1.5 (1.47 to 1.52) |  | 1.35 (1.32 to 1.37) | 1.38 (1.36 to 1.41) |
|  | Palliative care (QOF) | 2.29 (2.25 to 2.34) | 2.3 (2.25 to 2.35) |  | 1.83 (1.83 to 1.83) | 1.86 (1.82 to 1.9) |  | 1.42 (1.4 to 1.44) | 1.49 (1.46 to 1.53) |
|  | Bipolar & schizophrenia (GPES) | 1.23 (1.17 to 1.29) | 1.14 (1.07 to 1.21) |  | 1.2 (1.2 to 1.2) | 1.17 (1.07 to 1.27) |  | 1.19 (1.12 to 1.27) | 0.95 (0.84 to 1.07) |
|  | Coronary heart disease (GPES) | 0.97 (0.95 to 0.98) | 0.94 (0.93 to 0.96) |  | 1 (1 to 1) | 0.97 (0.95 to 0.98) |  | 1 (0.99 to 1.02) | 0.98 (0.97 to 1) |
|  | Epilepsy (GPES) | 1.13 (1.08 to 1.18) | 1.18 (1.14 to 1.23) |  | 1.14 (1.14 to 1.14) | 1.12 (1.07 to 1.17) |  | 1.15 (1.1 to 1.2) | 1.11 (1.04 to 1.17) |
|  | Learning dis. & Downs (GPES) | 1.44 (1.28 to 1.62) | 1.47 (1.32 to 1.64) |  | 1.47 (1.47 to 1.47) | 1.23 (1.05 to 1.45) |  | 1.34 (1.09 to 1.66) | 1.02 (0.78 to 1.34) |
|  | Liver cirrhosis (GPES) | 1.8 (1.69 to 1.91) | 1.81 (1.69 to 1.94) |  | 1.58 (1.58 to 1.58) | 1.56 (1.42 to 1.71) |  | 1.43 (1.29 to 1.59) | 1.31 (1.11 to 1.54) |
|  | Stroke & TIA (GPES) | 1.12 (1.1 to 1.14) | 1.11 (1.09 to 1.13) |  | 1.1 (1.1 to 1.1) | 1.08 (1.07 to 1.1) |  | 1.09 (1.08 to 1.11) | 1.08 (1.06 to 1.1) |
|  | Chronic respiratory disease (HES) | 1.42 (1.39 to 1.45) | 1.47 (1.44 to 1.49) |  | 1.35 (1.35 to 1.35) | 1.42 (1.39 to 1.45) |  | 1.24 (1.22 to 1.26) | 1.31 (1.28 to 1.34) |
|  | Cardiovascular disease (HES) | 1.46 (1.43 to 1.48) | 1.45 (1.42 to 1.47) |  | 1.39 (1.39 to 1.39) | 1.44 (1.41 to 1.46) |  | 1.32 (1.31 to 1.34) | 1.41 (1.38 to 1.43) |
|  | Chronic kidney disease (QOF only) | 0.99 (0.98 to 1.01) | 1.01 (0.99 to 1.02) |  | 0.98 (0.98 to 0.98) | 1.01 (0.99 to 1.03) |  | 1 (0.98 to 1.01) | 1.06 (1.04 to 1.08) |
|  | Chronic kidney disease (HES) | 1.41 (1.39 to 1.43) | 1.48 (1.46 to 1.5) |  | 1.35 (1.35 to 1.35) | 1.42 (1.4 to 1.44) |  | 1.25 (1.24 to 1.27) | 1.32 (1.3 to 1.35) |
|  | Dementia (HES or QOF) | 2.91 (2.87 to 2.96) | 2.81 (2.76 to 2.86) |  | 2.89 (2.89 to 2.89) | 2.56 (2.52 to 2.6) |  | 2.44 (2.41 to 2.47) | 2 (1.96 to 2.03) |
|  | Diabetes (QOF only) | 1.12 (1.09 to 1.15) | 1.15 (1.12 to 1.18) |  | 1.1 (1.1 to 1.1) | 1.12 (1.08 to 1.15) |  | 1.05 (1.02 to 1.08) | 1.07 (1.03 to 1.11) |
|  | Diabetes (HES) | 1.32 (1.3 to 1.34) | 1.3 (1.28 to 1.32) |  | 1.26 (1.26 to 1.26) | 1.25 (1.23 to 1.26) |  | 1.17 (1.15 to 1.18) | 1.17 (1.15 to 1.19) |
|  | Clinically vulnerable (NIMS) | 0.68 (0.67 to 0.69) | 0.58 (0.57 to 0.59) |  | 0.48 (0.48 to 0.48) | 0.42 (0.42 to 0.43) |  | 0.37 (0.37 to 0.38) | 0.29 (0.29 to 0.29) |
|  | Care home resident (GPES) | 1.62 (1.58 to 1.66) | 1.62 (1.58 to 1.66) |  | 1.54 (1.54 to 1.54) | 1.52 (1.48 to 1.56) |  | 1.36 (1.34 to 1.38) | 1.29 (1.26 to 1.32) |
| **Smoking status** | |  |  |  |  |  |  |  |  |
|  | Never smoker (NHSD) ^ref^ |  |  |  |  |  |  |  |  |
|  | Current smoker (NHSD) | 1.54 (1.5 to 1.58) | 1.39 (1.36 to 1.43) |  | 1.39 (1.39 to 1.39) | 1.24 (1.2 to 1.29) |  | 1.18 (1.13 to 1.22) | 1.13 (1.07 to 1.18) |
|  | Ex-smoker (NHSD) | 1.05 (1.03 to 1.06) | 0.96 (0.95 to 0.97) |  | 0.98 (0.98 to 0.98) | 0.93 (0.92 to 0.94) |  | 0.95 (0.94 to 0.96) | 0.93 (0.91 to 0.94) |
|  | Non-smoker (NHSD) | 1 (0.96 to 1.04) | 0.93 (0.9 to 0.97) |  | 0.98 (0.98 to 0.98) | 0.93 (0.89 to 0.96) |  | 0.95 (0.92 to 0.97) | 0.93 (0.89 to 0.97) |
| **Wave & COVID-19 & Vaccination status** | |  |  |  |  |  |  |  |  |
|  | Wave 1 & 0-4 weeks | 32.21 (30.88 to 33.6) | 38.13 (36.76 to 39.54) |  | 19.91 (19.91 to 19.91) | 25.3 (24.38 to 26.26) |  | 12.22 (11.79 to 12.66) | 15.78 (15.14 to 16.45) |
|  | Wave 1 & 4+ weeks | 3.89 (3.62 to 4.19) | 4.72 (4.4 to 5.05) |  | 3.45 (3.45 to 3.45) | 3.59 (3.34 to 3.86) |  | 2.54 (2.4 to 2.68) | 3 (2.77 to 3.25) |
|  | Wave 2 & 0-4 weeks | 30.94 (29.89 to 32.03) | 43 (41.66 to 44.38) |  | 21.95 (21.95 to 21.95) | 30.22 (29.24 to 31.23) |  | 14.84 (14.41 to 15.28) | 19.77 (19.04 to 20.53) |
|  | Wave 2 & 4+ weeks | 5.77 (5.45 to 6.1) | 7.09 (6.69 to 7.51) |  | 4.74 (4.74 to 4.74) | 6.96 (6.58 to 7.36) |  | 3.49 (3.33 to 3.66) | 5.28 (4.92 to 5.66) |
|  | Wave 3 & 0-4 weeks | 27.36 (24.34 to 30.75) | 40.23 (35.97 to 44.99) |  | 21.4 (21.4 to 21.4) | 34.12 (29.76 to 39.12) |  | 20.64 (18.21 to 23.4) | 33.18 (27.95 to 39.39) |
|  | Wave 3 & 4+ weeks | 4.39 (3.88 to 4.96) | 4.52 (3.91 to 5.22) |  | 3.89 (3.89 to 3.89) | 4.07 (3.43 to 4.82) |  | 3.2 (2.79 to 3.66) | 4.5 (3.62 to 5.59) |
|  | 1st vaccine date | 0.76 (0.74 to 0.78) | 0.92 (0.89 to 0.95) |  | 0.83 (0.83 to 0.83) | 0.95 (0.92 to 0.98) |  | 1.11 (1.08 to 1.14) | 1.32 (1.27 to 1.37) |
|  | Wave 2 & 0-4 weeks & 1st vaccine | 0.8 (0.74 to 0.87) | 0.79 (0.74 to 0.85) |  | 0.85 (0.85 to 0.85) | 0.88 (0.82 to 0.94) |  | 0.78 (0.74 to 0.83) | 0.84 (0.78 to 0.91) |
|  | Wave 3 & 0-4 weeks & 1st vaccine | 0.3 (0.26 to 0.34) | 0.27 (0.24 to 0.3) |  | 0.34 (0.34 to 0.34) | 0.31 (0.27 to 0.36) |  | 0.25 (0.22 to 0.28) | 0.27 (0.23 to 0.32) |
|  | Wave 2 & 4+ weeks & 1st vaccine | 0.49 (0.44 to 0.54) | 0.43 (0.39 to 0.48) |  | 0.56 (0.56 to 0.56) | 0.48 (0.43 to 0.53) |  | 0.6 (0.55 to 0.64) | 0.48 (0.42 to 0.54) |
|  | Wave 3 & 4+ weeks & 1st vaccine | 0.6 (0.53 to 0.68) | 0.59 (0.51 to 0.69) |  | 0.62 (0.62 to 0.62) | 0.72 (0.6 to 0.85) |  | 0.62 (0.54 to 0.71) | 0.57 (0.46 to 0.72) |
