# Supplementary material for: Life lost due to the COVID-19 pandemic: A model-based cohort analysis of mortality displacement in the registered population of England
Source: PLoS One. 2026 May 8;21(5):e0348575. doi: 10.1371/journal.pone.0348575 (PMC13155604; doi:10.1371/journal.pone.0348575)
Supplement: S3 Table — (DOCX) [file pone.0348575.s004.docx]

**Table S3 – Median weeks (and interquartile range) of distribution for displacement of mortality by age group and sex, 65 years or older, England**

| **Age Group** | **Females** | **Males** |
| --- | --- | --- |
| 65-69 | 749 (26-2016) | 515 (55-1362) |
| 70-74 | 549 (159-1210) | 372 (126-821) |
| 75-79 | 351 (164-657) | 247 (122-455) |
| 80-84 | 178 (96-317) | 137 (71-231) |
| 85-89 | 100 (54-162) | 83 (41-128) |
| 90+ | 74 (43-102) | 61 (33-88) |
| 65+ | 250 (76-832) | 228 (73-653) |
