# Supplementary material for: Life lost due to the COVID-19 pandemic: A model-based cohort analysis of mortality displacement in the registered population of England
Source: PLoS One. 2026 May 8;21(5):e0348575. doi: 10.1371/journal.pone.0348575 (PMC13155604; doi:10.1371/journal.pone.0348575)
Supplement: S4 Table — (DOCX) [file pone.0348575.s005.docx]

**Table S4 – Median weeks (and interquartile range) of distribution for displacement of mortality by age group and wave, 65 years or older, England**

| **Age Group** | **Wave 1** | **Wave 2** | **Wave 3** |
| --- | --- | --- | --- |
| 65-69 | 1241 (617-2304) | 1657 (930-2741) | 316 (-96-1135) |
| 70-74 | 605 (312-1118) | 1011 (557-1625) | 313 (77-745) |
| 75-79 | 321 (199-555) | 510 (278-814) | 228 (101-430) |
| 80-84 | 171 (125-270) | 240 (142-384) | 115 (52-209) |
| 85-89 | 105 (81-134) | 125 (86-196) | 58 (28-109) |
| 90+ | 74 (54-91) | 83 (54-113) | 50 (30-81) |
| 65+ | 182 (94-491) | 467 (156-1247) | 187 (41-578) |
