# Supplementary material for: Life lost due to the COVID-19 pandemic: A model-based cohort analysis of mortality displacement in the registered population of England
Source: PLoS One. 2026 May 8;21(5):e0348575. doi: 10.1371/journal.pone.0348575 (PMC13155604; doi:10.1371/journal.pone.0348575)
Supplement: S5 Table — (DOCX) [file pone.0348575.s006.docx]

**Table S5 – Estimated percentage (%) of people aged 65 or older who died with COVID-19 by expected length of survival had they not had the disease, by sex, 27 March 2020 to 30 December 2022**

|  | **1 year**  **or less** | **More than a year up to three years** | **More than three years up to five years** | **More than five years** |
| --- | --- | --- | --- | --- |
| Females | 22·0 | 36·0 | 13·8 | 28·2 |
| Males | 24·7 | 33·8 | 13·6 | 27·9 |
| Total | 23·5 | 34·8 | 13·7 | 28·0 |
