# Supplementary figures and images for: Life lost due to the COVID-19 pandemic: A model-based cohort analysis of mortality displacement in the registered population of England
Source: PLoS One. 2026 May 8;21(5):e0348575. doi: 10.1371/journal.pone.0348575 (PMC13155604; doi:10.1371/journal.pone.0348575)

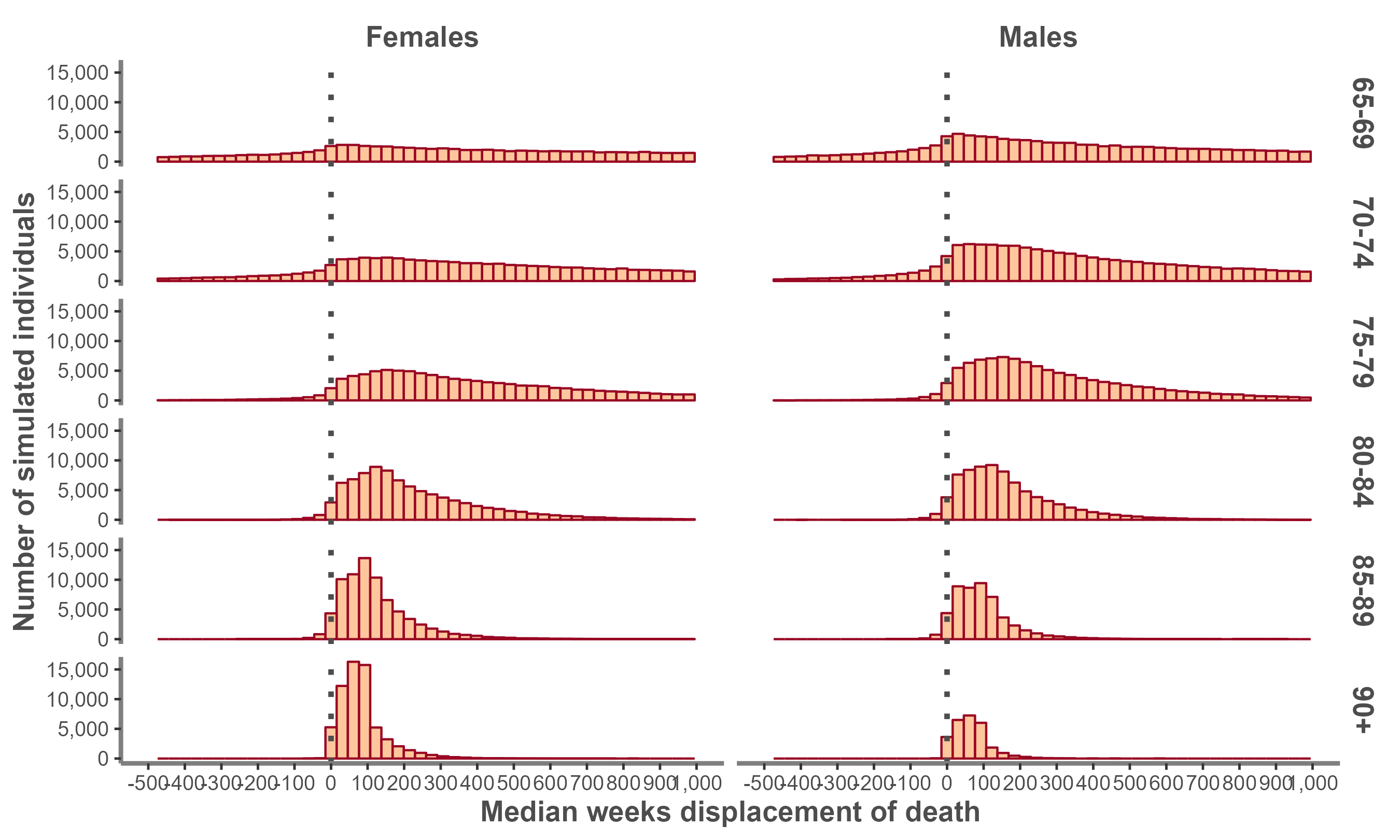

Supplement: S1 Fig — (TIFF) [file pone.0348575.s007.tiff]

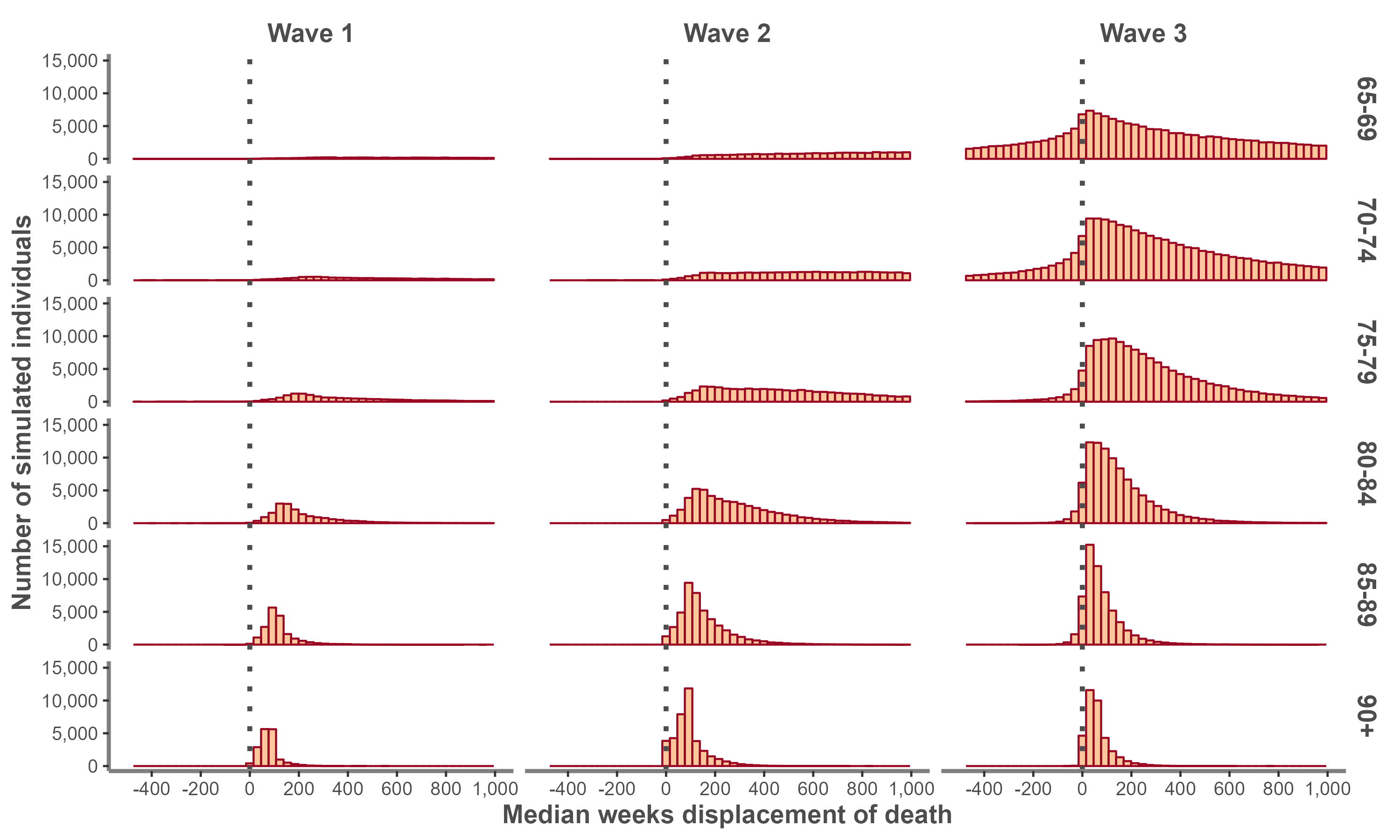

Supplement: S2 Fig — (TIFF) [file pone.0348575.s008.tiff]
